# Eye morphogenesis in the blind Mexican cavefish

**DOI:** 10.1242/bio.059031

**Published:** 2021-10-28

**Authors:** Lucie Devos, François Agnès, Joanne Edouard, Victor Simon, Laurent Legendre, Naima El Khallouki, Sosthène Barbachou, Frédéric Sohm, Sylvie Rétaux

**Affiliations:** 1 Paris-Saclay Institute of Neuroscience, CNRS, Université Paris-Saclay, 91198 Gif sur Yvette, France; 2 AMAGEN, CNRS, INRA, Université Paris-Saclay, 91198, Gif sur Yvette, France

**Keywords:** *Zic1*, CRISPR/Cas9 knock-in, Live imaging, Cell behaviours, Optic vesicle, Retinal pigmented epithelium, Evo-devo

## Abstract

The morphogenesis of the vertebrate eye consists of a complex choreography of cell movements, tightly coupled to axial regionalization and cell type specification processes. Disturbances in these events can lead to developmental defects and blindness. Here, we have deciphered the sequence of defective events leading to coloboma in the embryonic eye of the blind cavefish of the species *Astyanax mexicanus*. Using comparative live imaging on targeted enhancer-trap *Zic1:hsp70:GFP* reporter lines of both the normal, river-dwelling morph and the cave morph of the species, we identified defects in migratory cell behaviours during evagination that participate in the reduced optic vesicle size in cavefish, without proliferation defect. Further, impaired optic cup invagination shifts the relative position of the lens and contributes to coloboma in cavefish. Based on these results, we propose a developmental scenario to explain the cavefish phenotype and discuss developmental constraints to morphological evolution. The cavefish eye appears as an outstanding natural mutant model to study molecular and cellular processes involved in optic region morphogenesis.

## Abstract (precis)

**Summary:** We show that the developing embryonic eye of the blind Mexican cavefish suffers multiple tissue morphogenesis and cell behaviour defects that lead to coloboma.

## INTRODUCTION

The morphogenesis of vertebrate eyes follows a complex choreography of cell movements, from a flat neural plate to a spherical multi-layered structure. It is advantageously investigated on teleost models, amenable to live imaging (Cavodeassi, 2018).

At the end of gastrulation, the ‘eyefield’ is specified in the anterior neural plate, surrounded by prospective telencephalon, hypothalamus and diencephalon (Varga et al., 1999; Woo and Fraser, 1995; Woo et al., 1995). The first step of eye formation is the lateral evagination of optic vesicles (OV) (England et al., 2006; Ivanovitch et al., 2013; Rembold et al., 2006). Vesicles then elongate due to an anterior/nasal flow of cells – a process called ‘extended evagination’ (Kwan et al., 2012) – and get separated from the neural keel by the anterior-wards progression of a posterior furrow (England et al., 2006). Cells from the inner OV leaflet then migrate around the rim of the eye ventricle into the lens facing neuroepithelium through the ‘rim movement’ (Heermann et al., 2015; Kwan et al., 2012). Cells fated to the retinal pigmented epithelium (RPE) expand and flatten to cover the back of the retina (Cechmanek and McFarlane, 2017; Heermann et al., 2015). Together with basal constriction of lens-facing epithelial cells (Martinez-Morales et al., 2009; Nicolas-Perez et al., 2016), these movements lead to optic cup (OC) invagination, and also to the formation of the optic fissure, which needs to close to have a proper, round eye (Gestri et al., 2018). Finally, the entire forebrain rotates anteriorly, bringing the fissure in its final ventral position. Hence, cells initially located in the dorsal or ventral OV contribute to the nasal or temporal quadrant of the retina, respectively (Picker et al., 2009) (Fig. S1). Failure to complete any of these steps can lead to vision defects; for example, failure to close the optic fissure is termed coloboma.

*Astyanax mexicanus* is a teleost that arises in two morphs: eyed river-dwelling morphs and blind cave-dwelling morphs. Although eyes are absent in adult cavefish, they first develop in embryos before degenerating during larval stages. The embryonic cavefish eyes display multiple abnormalities: the OVs are short (Alunni et al., 2007), the OC and lens are small (Hinaux et al., 2015, 2016; Yamamoto and Jeffery, 2000) and the ventral OC is severely reduced, with the fissure wide open and a coloboma phenotype (Pottin et al., 2011; Yamamoto et al., 2004). Cavefish exhibit modifications in morphogen expression, which have been linked to their eye defects. Accordingly, overexpression of *Shh* in surface fish shortens its optic cups and triggers lens apoptosis, while inhibition of Fgf signalling in cavefish restores the ventral retina (Hinaux et al., 2016; Pottin et al., 2011; Torres-Paz et al., 2019; Yamamoto et al., 2004).

Because of these variations, cavefish embryos are remarkable natural mutant models to study eye development, beyond the mechanisms of eye degeneration. Here, we sought to understand cavefish embryonic eye morphogenetic defects as well as the mechanisms of eye morphogenesis in general. We generated *Astyanax* CRISPR/Cas9-targeted enhancer trap *Zic1:hsp70:GFP* reporter lines to perform comparative live imaging and uncover the morphogenetic processes and cellular behaviours leading to cavefish coloboma.

## RESULTS AND DISCUSSION

### Establishing *Zic1:hsp70:GFP* surface fish (SF) and cavefish (CF) reporter lines

**Fig. 1.**
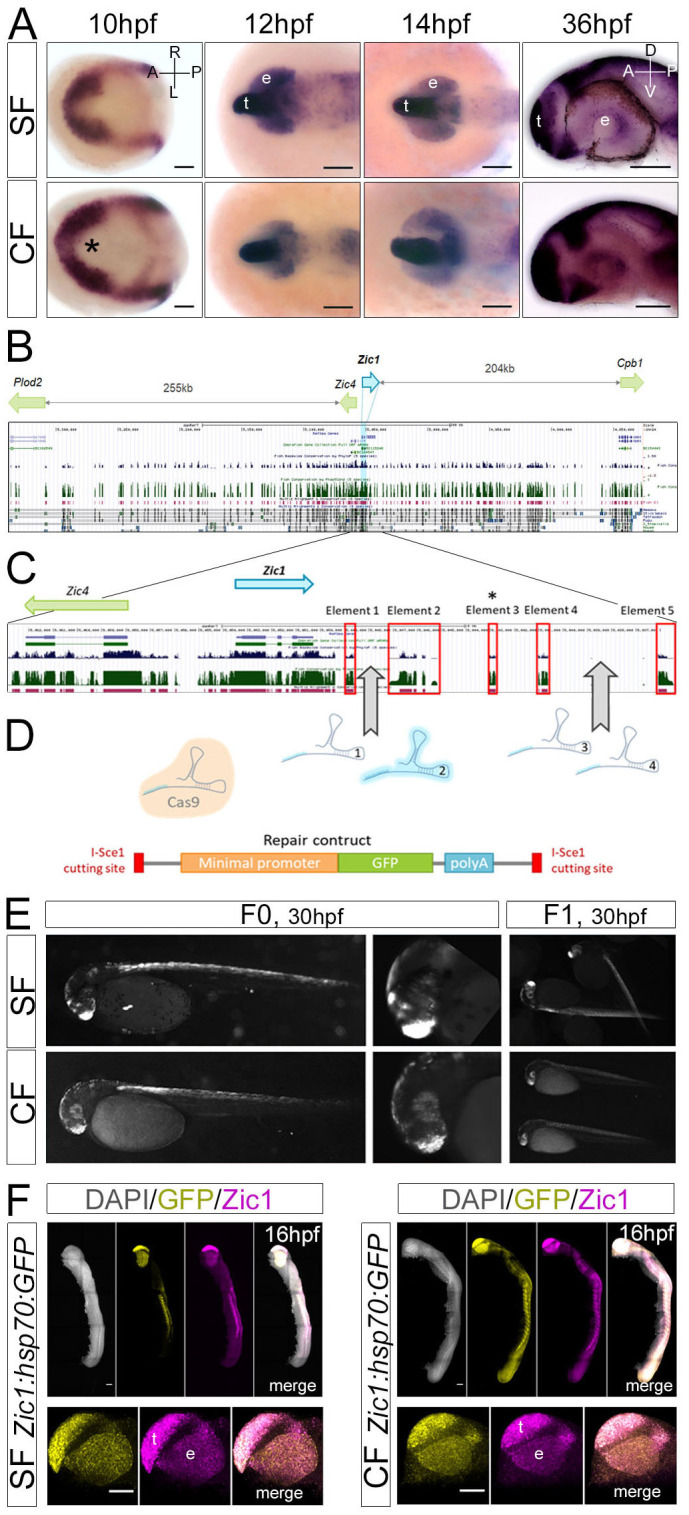
***Zic1:hsp70:GFP* reporter lines.** (A) *Zic1* expression time-course. Asterisk: larger indentation in CF eyefield. (B) Zebrafish *Zic1* genomic region in UCSC genome browser (2010 assembly). Green/blue peaks and magenta/black elements correspond to high conservation. (C) Close-up on *Zic1*. Red boxes highlight conserved elements; element 3 is not conserved in *Astyanax* (asterisk). (D) sgRNA designed to target the low-conservation regions between elements 1/2, and 4/5. SgRNA2 (pale blue) efficiently generated cuts. It was co-injected together with Cas9 protein and a linear repair construct (*Hsp70:GFP*). (E) *Zic1-like* GFP fluorescence in mosaic F0 s and stable F1 s. (F) Double-fluorescent *in situ* hybridization at 16 hpf for *Zic1* (magenta) and *GFP* (yellow). Lateral views. The transgene recapitulates endogenous *Zic1* pattern, both for SF and CF lines. Top panels show entire embryos, bottom panels show close-ups on the head. t, telencephalon; e, eye. Scale bars: 100 µm.

After an *in situ* hybridization mini-screen for genes labelling the optic region from neural plate stage (10 hpf) until at least 30 hpf (Fig. S2A), *Zic1* was chosen for its early and persistent expression (Fig. 1A; Fig. S2B), although its pattern was larger than the optic region.

We used a targeted enhancer-trap strategy into the *Zic1* locus, so that the GFP reporter insertion site would be similar in CF and SF lines and avoid positional effects, which is crucial for comparative purposes. The large *Zic1* genomic region was examined. In both zebrafish and *Astyanax* genomes (McGaugh et al., 2014), *Zic1* and *Zic4* were located in a head-to-head configuration in a gene desert containing many fish-conserved, partly tetrapod-conserved elements (Fig. 1B,C). We targeted a reporter construct into *Zic1* downstream region using CRISPR/Cas9, similarly to the approach used in (Kimura et al., 2014). We reasoned that using NHEJ (non-homologous end-joining) DNA repair mechanism-based strategy, the preferred repair mechanism in fish embryos (Hagmann et al., 1998), would maximise integration efficiency. Eggs were co-injected with sgRNA2, Cas9 protein and a linearised minimal promoter *hsp70:GFP* repair construct (Fig. 1D). The method yielded good results; its limited efficiency being compensated by the possibility of pattern-based fluorescence screening in F0 embryos (Fig. 1E). Genomic analyses confirmed the proper insertion of the transgene at the targeted site, although some structural differences existed between lines (Fig. S3). The insertion being based upon non-conservative NHEJ mechanism, these variations are likely due to indels/duplications differences, which may slightly affect nearby regulatory sequences. However, such variations remain anecdotal compared to those observed between lines generated by traditional transgenesis techniques (like Tol2-transgenesis) (Elipot et al., 2014; Hinaux et al., 2015; Stahl et al., 2019), validating our approach as a valuable tool to follow gene expression in *Astyanax*. Double-fluorescent *in situ* hybridization demonstrated that the *GFP* reporter fully recapitulated the endogenous *Zic1* pattern at the stages of interest (Fig. 1F).

CRISPR/Cas9 was used previously in surface *Astyanax* to target *Oca2* and confirm the role of *Oca2* in pigmentation control (Klaassen et al., 2018). Here, we successfully used the CRISPR/Cas9 technology in this emergent model species to generate identical reporter lines in the two morphotypes, and in a targeted genome edition perspective.

### Comparing eye morphogenesis in surface fish and cavefish through live-imaging

**Fig. 2.**
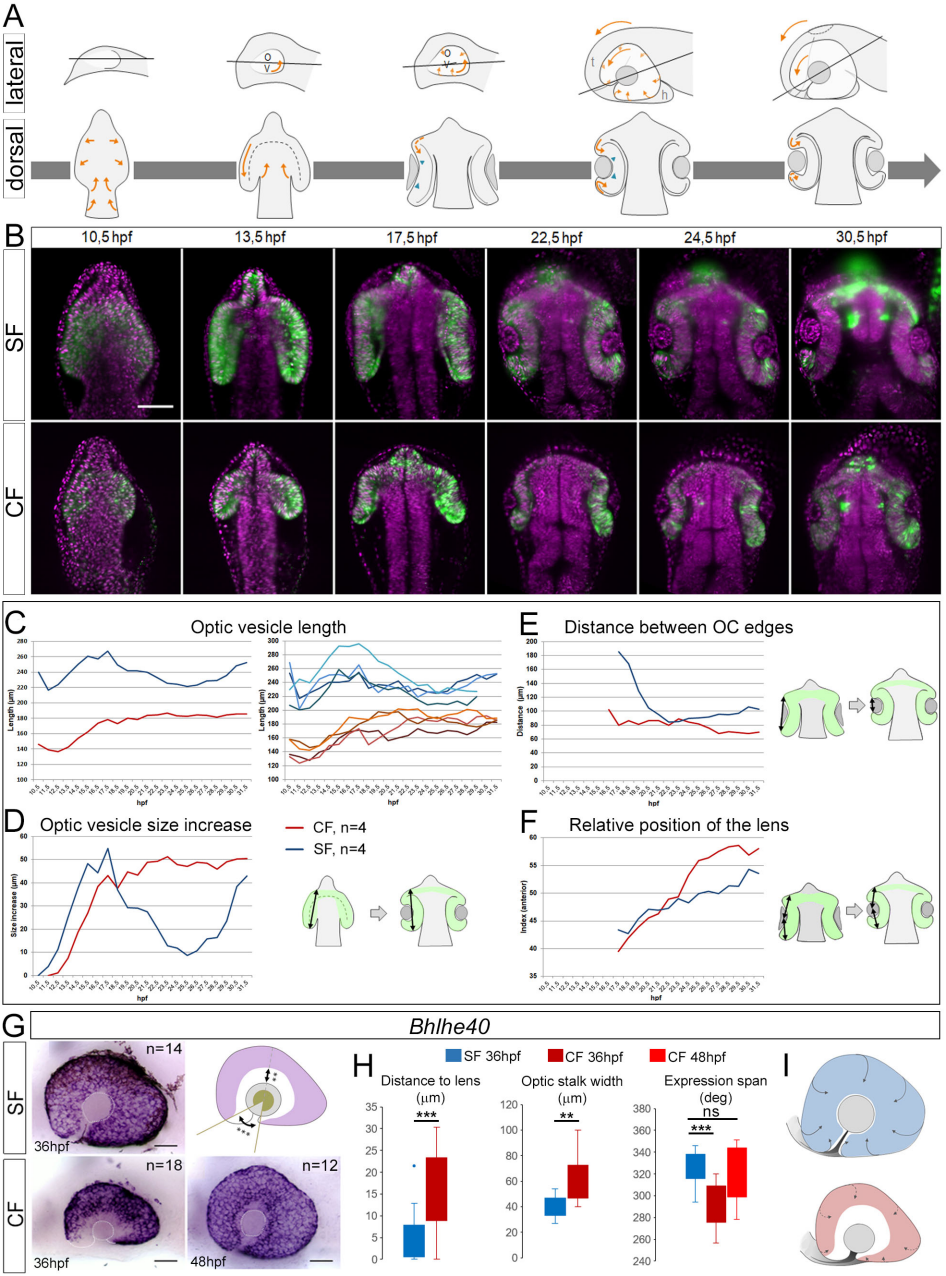
**Eye morphogenesis.** (A) Schematic drawings of the main steps of fish eye morphogenesis. Orange arrows, cell/tissue movements; green arrowheads, initiation of basal constriction; grey line, optical section plane shown in B (follows an optic stalk-to-lens centre axis and accompanies the final anterior rotation). All measures in C–F were performed on these planes. (B) Still images of time-lapse acquisitions from 10.5 hpf to 30.5 hpf on SF (top) and CF (bottom) *Zic1:hsp70:GFP* lines (green: GFP; magenta: nuclear mCherry). Representative steps of eye morphogenesis illustrating CF/SF differences are shown. Dorsal views, anterior to the top. (C–F) Measurements, as illustrated on drawings. (C) OV length. The left graph shows the mean of *n*=4 eyes in each morph; the right graph displays the trajectories of individual eyes, showing reproducibility. (D) OV size increase. (E) Distance between OC edges. (F) Position of lens relative to anterior OV. (G-I) *Bhlhe40* expression. The top-right scheme shows measures taken in H. (I) Drawings illustrating comparative RPE spreading in the two morphs. Numbers of embryos analysed are indicated in G. Mann–Whitney test: **P<0.01; ***P<0.001.

Live-imaging was performed on a light-sheet microscope on *Zic1:hsp70:GFP* embryos co-injected with H2B-mCherry mRNA to follow cell nuclei, from ∼10.5 hpf to 24–30 hpf (Fig. 2**;** Movies 1–2).

For analysis, we chose a plane crossing the middle of the lens and the optic stalk (Fig. 2A, lines), to follow the anterior rotation of the eye. Overall, optic morphogenesis in SF recapitulated the events described in zebrafish, while in CF the movements were conserved but their relative timing and extent appeared different.

#### Evagination and elongation

The cavefish OVs were half-shorter than the SF OVs from the beginning of evagination onwards (Fig. 2A–C). Elongation progressed at the same pace as in SF until 17.5 hpf (Fig. 2C). However, while OV length decreased between 17.5–25.5 hpf in SF due to invagination, elongation continued at slower pace until 25.5 hpf in CF (Fig. 2C,D). Moreover, SF OVs remained closely in contact with the neural tube, while in CF they first started growing away before getting back closer between 18.5–21.5 hpf (Fig. 2B). Throughout development, the width of the optic stalk was similar in the two morphs (Fig. S4), despite an initially smaller size in CF due to the smaller OVs.

Since elongation proceeds at a similar rate in CF and SF until 17.5 hpf, the shorter size of the cavefish OV (Alunni et al., 2007; Strickler et al., 2001) seems principally due to the small size of the initial eyefield (Agnès et al., 2021 preprint). Albeit smaller, CF OVs seem correctly patterned in their future naso-temporal axis, according to *FoxG1*/*FoxD1* markers at 13.5 hpf (Hernandez-Bejarano et al., 2015). Then, after initial evagination and patterning of small OVs, morphogenesis proceeds with the extended evagination, whereby cells from the neural tube continue entering the OV to contribute exclusively to the ventro-nasal part of the eye (Gordon et al., 2018; Kwan et al., 2012). Our measurements suggest that this step proceeds normally in CF. This could partially compensate the originally small size of the eyefield/OV, but only in the nasal part, while the temporal part would remain affected in size.

#### Invagination and lens formation

In both morphs, the posterior end of the OVs started curling back around 15.5 hpf and the lens was identifiable as an ectodermal thickening at 17.5 hpf (Fig. 2B; Movies 1,2), in a central position relative to the antero-posterior extension of the OV (Fig. 2B,F). Then, in SF, invagination quickly brought the OC edges in contact with the lens (Fig. 2B,E). In CF, despite initially harbouring some curvature, the OC edges remained flat (Fig. 2B,E; Movie 2) and continued to elongate while the lens remained static, therefore shifting the lens position anteriorly (Fig. 2B,F). The posterior OC showed slow/reduced curling, which sometimes led to separation from the lens. Eventually, the posterior (prospective dorsal) OC curved and contacted the lens (Movie 2; Fig. 2B), but remained shallower, with small bulging lens.

Although invagination in CF seems to start properly between 15.5–19.5 hpf, it progresses poorly so that OCs remain elongated. Such timing is reminiscent of the two steps described for OC invagination in zebrafish: basal constriction initiates the primary folding between 18–20 hpf (18–22 ss), followed by rim movement which brings the presumptive retina from the inner OV leaflet into the lens-facing epithelium between 20–24 hpf (Heermann et al., 2015; Nicolas-Perez et al., 2016; Sidhaye and Norden, 2017). In *Astyanax*, 18ss corresponds to ∼16.5 hpf (Hinaux et al., 2011), hence initial basal constriction leading to initiation of OC invagination may be partly conserved in cavefish. However, the prolonged extension and the weak curvature of the OVs suggest that the rim movement is probably impaired. We suggest that a continuous flow of cells entering the retina leads to its elongation, in the absence of efficient invagination. The latter is weak but not absent in CF, as the posterior OC still manages to contact the lens, at later stages. The defective rim movement might be due to various causes, including defects in the basal membrane or failure to establish proper focal adhesion as in the *ojoplano* medaka mutant (Martinez-Morales et al., 2009; Nicolas-Perez et al., 2016; Sidhaye and Norden, 2017). Alternatively, active migration could be altered by extrinsic signals, as in BMP overexpression experiments where the cell flow toward the lens-facing epithelium is reduced (Heermann et al., 2015). The various morphogen modifications known in cavefish, and the fact that the ventral eye can be restored by delaying the onset of Fgf signalling in CF to match the SF timing (Pottin et al., 2011), support this possibility.

Spreading and migration of RPE cells are concomitant with the rim movement and may contribute to it as a driving force (Cechmanek and McFarlane, 2017; Moreno-Marmol et al., 2018). In 36 hpf SF embryos, the RPE marker *Bhlhe40* was expressed all around the eye, often contacting the lens (Fig. 2GH), which we took as an indicator of the correct engulfment of the retina by the migrating RPE. Conversely, in CF, *Bhlhe40*-positive cells were further away from the lens, with a wider ventral gap possibly corresponding to wider optic fissure opening, suggesting reduced or delayed retina covering by RPE cells (Fig. 2G–I). At 48 hpf, however, the staining span was no longer different from the 36 hpf SF. These data show that RPE identity is maintained in CF eyes, yet its expansion movement to cover the whole retina is delayed compared to SF – reinforcing the notion that the rim movement is impaired in cavefish and that RPE spreading could contribute to invagination forces (Moreno-Marmol et al., 2021 preprint). Potentially, RPE spreading may also be involved in optic fissure closure, as suggested by the presence of coloboma upon impairment of the rim movement by *BMP4* overexpression in the OV (Heermann et al., 2015). Deficiency in RPE spreading might participate in the cavefish coloboma (Fig. 2I). Interestingly, the transplantation of a healthy SF lens into the CF OC rescues the eye as a structure, i.e. prevents lens-induced degeneration, but does not rescue coloboma (Yamamoto and Jeffery, 2000). This is consistent with our findings showing that improper closure of the fissure is autonomous to CF retinal tissues and results from defective morphogenetic movements.

The lens forms in proper place and time, in both morphs, relative to OC invagination onset. It is only later that the lens appears anteriorly-shifted in cavefish. This apparent displacement of the lens relative to the retina is not due to a movement of the lens itself, which remains fixed throughout morphogenesis (Greiling and Clark, 2009), attached to the overlying ectoderm from which it delaminates ∼22 hpf in *Astyanax* (Hinaux et al., 2017), but rather to persistent OV elongation. This suggests that proper initial interactions occur between the central OV and the lens to adjust their relative position and initiate OC invagination. In chick, the pre-lens ectoderm is required for OC invagination while the lens placode itself is dispensable (Hyer et al., 2003). In cavefish, such mechanisms could exist and lead to the initiation of OC folding, as we observed. Finally, the anterior-shifted position of the lens, due to elongation without invagination, explains how the lens is ventrally-displaced in the larval CF eye after the final anterior rotation movement (Fig. 2A,I).

Our live-imaging experiments suggest that, in CF, OVs are reduced in size after the initial evagination, elongation occurs properly, while invagination is transiently compromised. Next, we started addressing cellular behaviours that may underlie these phenotypes, focusing on the small size of the evaginating OVs. We tracked cells during evagination, between 11.5–13 hpf (1 h 40 min, 40 movie frames).

#### OV cells proliferation

**Fig. 3.**
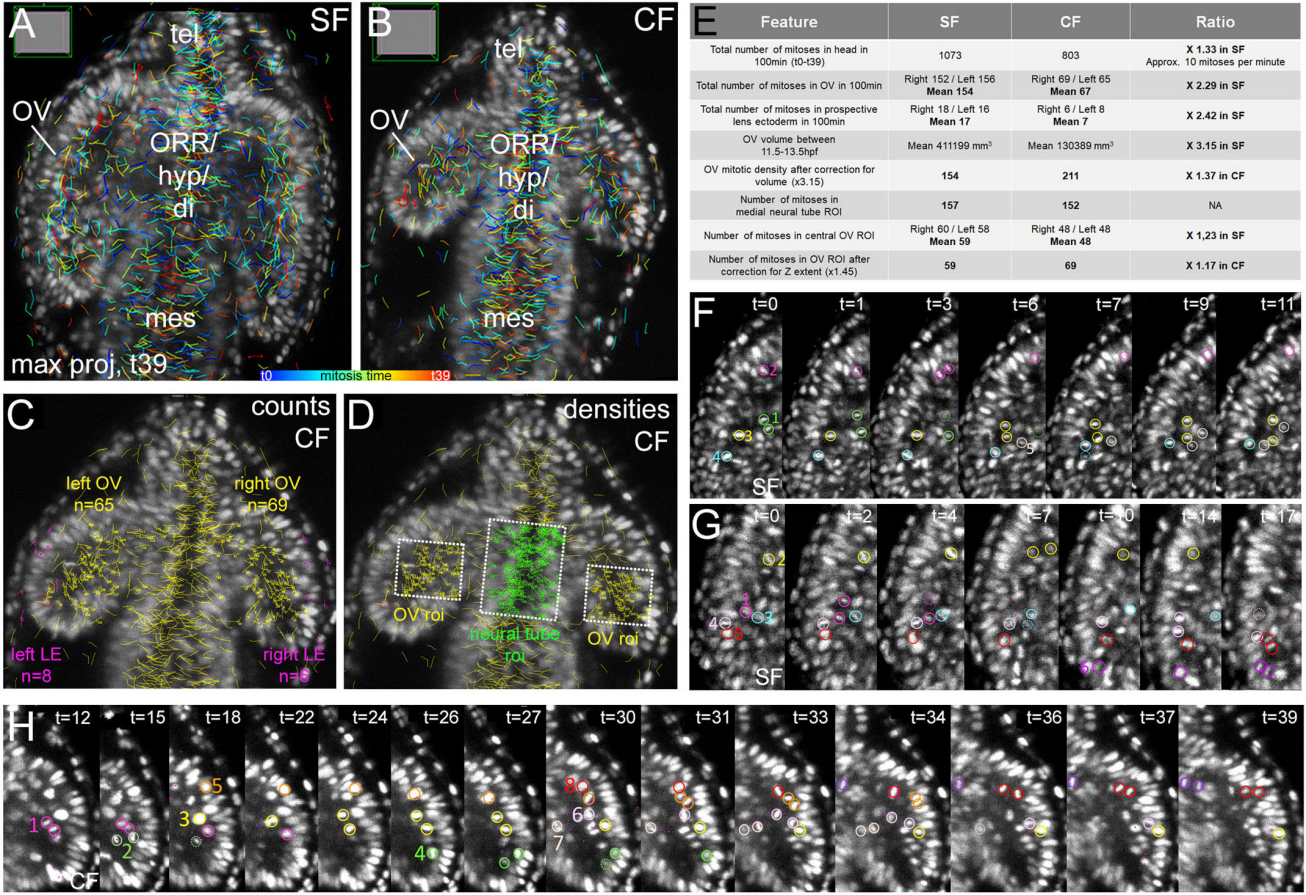
**Cell divisions.** (A,B) Mitotic embryos. Mitoses tracked during 100 min (40 time-steps*2.5 min) shown on maximum projection dorsal views at t=39 (end of movies) in SF (A) and CF (B). Colour code indicates division time (Fig. S5). tel/Telencephalon; mes/Mesencephalon; Optic Recess Region/Hypothalamus/Diencephalon contribute to the medial neural tube and cannot be delineated without molecular markers on this dorsal view. (C,D) Quantification, shown here on CF (Fig. S6). (C) Counts. Mitoses with yellow and pink numbers belong to OVs and presumptive lens ectoderm, respectively. (D) Densities. Regions of interest (ROI) of identical size were analysed, in OVs (yellow) or medial neural tube (green). (E) SF/CF comparisons. (F,G,H) Cell division behaviours are qualitatively indistinguishable between SF (F,G) and CF (H). Coloured circles help following individual nuclei (Fig. S7).

Division rates may account for size differences. To test this hypothesis, we reconstructed the complete mitotic pattern of the forebrain or head, in one CF and one SF embryo. Metaphase plates were searched manually and tracked at each time step through the depth of the embryos (Movies 3–6; Fig. 3A,B). A total of 1073 and 803 cell divisions were annotated in SF and CF, respectively, during the 100 min studied. Hence, the proliferation rate is ∼10 mitoses per minute in the fast-neurulating fish forebrain. In both morphs, mitoses were evenly distributed in time and space – disregarding their tendency to occur close to ventricles (below and Fig. S5).

In both OVs and prospective lenses (ectoderm in direct contact with OV), the left/right symmetry of mitoses distributions/numbers was excellent, suggesting that the mitotic landscape was accurately reconstituted. There, we found about twice more cell divisions in SF than in CF (Fig. 3A–E; Fig. S6). Conversely, division numbers were similar in SF and CF in a medial neural tube region of defined size used as control (Fig. 3D,E). To compare division rates, mitoses numbers were normalised to OV volumes, in two different ways (Fig. 3C–E; Fig. S6). Unexpectedly, the normalised mitotic activity appeared slightly higher in cavefish OVs, suggesting that proliferative activity may tend to compensate for small eyefield size (Agnès et al., 2021 preprint). Further, these data suggest that a quantitative defect in proliferation does not participate in the establishment of OV size differences. To examine the possibility of a qualitative defect that may also account, directly or indirectly, to the cavefish phenotype, we next inspected cell division behaviours in the evaginating OVs. The mitotic behaviours of SF and CF optic cells were indistinguishable. The migration towards the ventricle (optic recess), the orienting/rotating behaviour of metaphasic plates before dividing in apical position, and the post-division integration of daughter cells into the neuroepithelium were systematically observed in both morphs (Movies 7,8; Fig. 3F–I; Fig. S7). These results rule out an early proliferative defect in CF OVs to explain their small size, and parallels studies at later stages which dismissed a role for defective proliferation during CF eye degeneration (Alunni et al., 2007; Strickler et al., 2002). Cavefish OVs also appear like an outstanding model to study developmental mechanisms controlling organ size and developmental robustness (Young et al., 2019).

Our study has clear limitations regarding only one embryo per morph being analysed. Of note, as shown in morphometric measurements in Fig. 2C (right panel), the global growth curves of the optic vesicles in different embryos (*n*=2 for each morphotype) and in the two eyes of a given embryo (hence *n*=4 CF eyes and *n*=4 SF eyes on the graph) are very similar and show little inter-individual or inter-eye variation. Hence, one can suppose that the proliferation analysis performed on one of each of these embryos is representative. In addition, there was an excellent left-right symmetry in the mitoses tracked in the two eyes of each embryo (Fig. 3E), which is also in favour of the quantification in the two eyes of a single embryo being a good proxy of the proliferative activity in SF and CF eyes in general.

#### OV cells trajectories

**Fig. 4.**
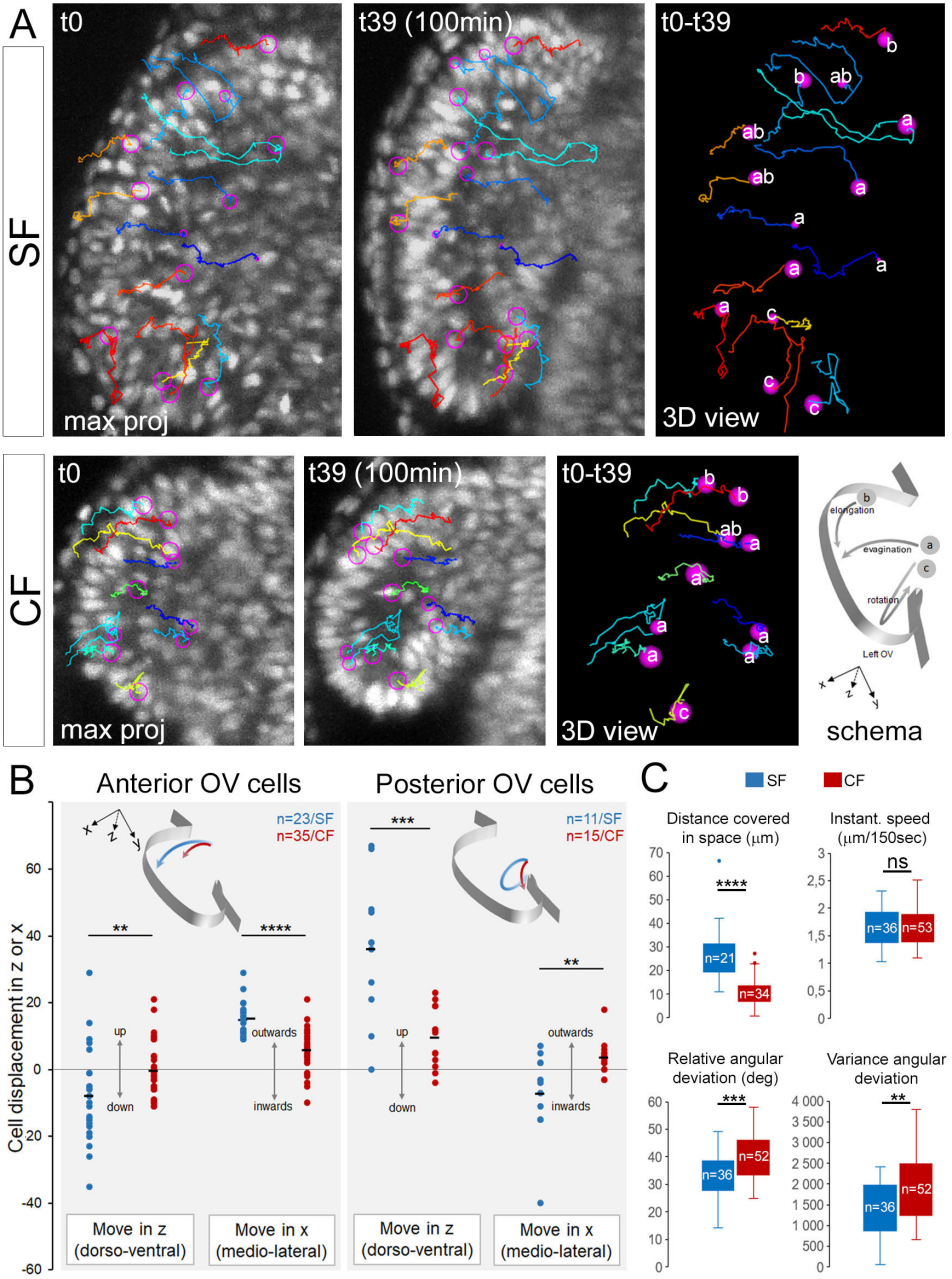
**Cell trajectories.** (A) Cell tracking and trajectories. Representative examples of cells tracked during 100 min, shown on maximum projection dorsal views at t=0 and t=39 (start/end of movies) and on 3D views. Individual cell tracks are in different colours, nuclei in pink circles. The bottom right schema illustrates the three main types of trajectories (a/evagination; b/elongation; c/rotation). (B) Quantifications of trajectories and directions followed by cells of the 2/3 anterior versus 1/3 posterior OV, in SF (blue) and CF (red). (C) Cell migration parameters. Mann–Whitney tests: *P<0.05; **P<0.01; ***P<0.001.

To test the possibility that defective migratory properties might contribute to small cavefish OVs, 24 SF and 44 CF cells were tracked between 11.5–13 hpf (Fig. 4). In SF, we observed markedly different types of trajectories depending on the initial position of cells. Namely, cells located in the two-thirds anterior OV showed a lateral-wards movement with a slight tendency to dive towards the ventral side, thus contributing to evagination (Fig. 4A,B). Some anterior cells also followed a posterior turn or had a strict antero-posterior trajectory, potentially contributing to elongation (Fig. 4A,B). Conversely, cells located in the OVs posterior third followed dorsal-wards and inwards paths, seemingly imposing a rotational movement to the posterior OV (Fig. 4A,B), possibly corresponding to the ‘pinwheel movement’ described in zebrafish (Kwan et al., 2012).

Most of these trajectories were impaired in CF (Fig. 4A,B). Anterior cells displayed reduced outwards movement and remained static in depth, showing reduced contribution to evagination. Posterior cells trajectories had less amplitude in the upwards direction and displayed outwards instead of inwards trajectories. In contrast, cells with posterior-wards trajectories contributing to elongation were observed in CF (Fig. 4A), in line with the proper elongation recorded above (Fig. 3). These analyses suggested that CF optic cells adopted improper behaviours in terms of trajectories during evagination.

We then compared kinetic parameters of cell migrations. The instantaneous speed and the total distance travelled by OV cells in the two morphs were similar (Fig. 4C), suggesting that the migrating apparatuses and capacities of CF cells were unaffected. However, the total displacement in space was shorter for CF cells, in line with above results on trajectories. To reconcile these seemingly contradictory observations, we measured deviation angles of trajectories between different time steps. We discovered a significant zigzagging, erroneous aspect of CF cells migration, as compared to the straighter paths of SF cells (Fig. 4C). This suggests that cavefish optic cells partly lacked or failed to respond to guidance and directionality cues.

### Conclusions

Thanks to genome-editing and live-imaging methods, we have started deciphering the morphogenetic and cellular processes underlying colobomatous eye development in cavefish. Our data pave the way for experiments analysing the defective molecular mechanisms in cavefish eye morphogenesis, using *Zic1:hsp70:GFP* knock-in lines and recently-developed embryology methods (Torres-Paz and Rétaux, 2021). They also illustrate how the very first steps of eye morphogenesis constitute an absolute developmental constraint to morphological evolution that cannot be circumvented, even in animals that eventually become eyeless adults (Durand, 1976; Rétaux and Casane, 2013; Stemmer et al., 2015; Wilkens, 2001). Our results help refine the step(s) in eye morphogenesis that are mandatory and constrained. In cavefish, the eyefield is specified and the evagination/elongation steps, corresponding to cell movements leading to the sorting of retinal versus adjacent telencephalic, preoptic and hypothalamic cells, do occur. It is only after the segregation between these differently fated cell populations that cavefish eye morphogenesis starts going awry, with a defective invagination process, soon followed by lens apoptosis and progressive degeneration of the entire eye. Therefore, our data support the idea that the first steps of eye morphogenesis constitute an absolute developmental constraint to morphological evolution. To our knowledge, the closest to a counter-example is the medaka mutant *eyeless*, a temperature-sensitive *rx3* mutant line in which OVs do not evaginate. However, the homozygous *eyeless* fish either die after hatching (Winkler et al., 2000), or, for the 1% that reach adulthood, are sterile probably due to anatomical hypothalamic or hypophysis defects (Ishikawa et al., 2001), confirming a strong developmental constraint on vertebrate eye morphogenesis.

## MATERIALS AND METHODS

### Animals

Laboratory stocks of *A. mexicanus* surface fish and cavefish were obtained in 2004 from the Jeffery laboratory at the University of Maryland. The surface fish were originally collected from San Solomon Spring, Texas, USA, and the cavefish are from the Pachón cave in Mexico. Surface fish are kept at 26°C and cavefish at 22°C. Natural spawns are induced after a cold shock (22°C over weekend) and a return to normal temperature for surface fish; cavefish spawns are induced by raising the temperature to 26°C. Embryos destined for *in situ* hybridization were collected after natural spawning, grown at 24°C and staged according to the developmental staging table (Hinaux et al., 2011) and fixed in 4% paraformaldehyde. After progressive dehydration in methanol, they were stored at −20°C. Embryos destined to transgenesis or live imaging were obtained by *in vitro* fertilization. Embryos were raised in an incubator until 1 month post fertilization for the surface fishes and 2 months post fertilization for the cavefish. They were kept at low density (15/20 per litre maximum) in embryo medium, in 1 litre plastic tanks with a soft bubbling behind the strainer. Larvae were fed from day 5 with paramecium and transitioned to artemia nauplii from day 10–15. Artemia were given twice a day except for the weekends (once a day) and carefully removed afterward to avoid polluting the medium. At least two-thirds of the medium were changed every day and dead larvae removed. After 1 month for the surface fish and 2 months for the cavefish, juveniles were taken to the fish facility where they were fed dry pellets (Skretting Gemma wean 0.3) and quickly moved to bigger tanks in order to allow their fast growth.

Animals were treated according to French and European regulations of animals in research. SR’ authorization for use of animals in research is 91–116, and Paris Centre-Sud Ethic committee authorization numbers are 2012–52 and 2012–56.

### *In situ* hybridization

Some cDNAs were available from our cDNA library: *Zic1* (FO290256), *Zic2a* (FO320762) and *Rx3* (FO289986); others were already cloned in the lab: *Lhx2* (EF175737) and *Lhx9* (EF175738) (Alunni et al., 2007); obtained from other labs (*Vax1*: Jeffery lab, University of Maryland; Yamamoto et al., 2004); or cloned for the purpose of this work in pGEMT-Easy (Promega). Vax2, forward primer: GGGCAAAACATGCGCGTTA; reverse primer CAGTAATCCGGGTCCACTCC. Bhlhe40, forward primer: GCACTTTCCCTGCGGATTTC; reverse primer: TGGAGTCTCGTTTGTCCAGC.

cDNAs were amplified by PCR, and digoxygenin-labelled riboprobes were synthesised from PCR templates. Embryos were rehydrated by graded series of EtOH/PBS, then for embryos older than 24 hpf, proteinase-K permeabilization at 37°C was performed for 36 hpf embryos only (10 µg/ml, 15 min) followed by a post-fixation step. Riboprobes were hybridised for 16 h at 65°C and embryos were incubated with anti-DIG-AP (Roche, dilution 1/4000) overnight at 4°C. Colorimetric detection with BCIP/NBT (Roche) was used. Mounted embryos were imaged on a Nikon Eclipse E800 microscope equipped with a Nikon DXM 1200 camera running under Nikon ACT-1 software. Brightness and contrast were adjusted using FIJI, some of the images used for illustration purpose were created from an image stack, using the extended depth of field function of Photoshop CS5. Area, distance and angle measurements were performed using FIJI (Schindelin et al., 2012).

### *In vitro* fertilization (IVF) and injections

Surface and cavefish were maintained in a room with shifted photoperiod (light: 4pm – 7am, L:D 15:11) in order to obtain spawns during the working day (*Astyanax* spawn at night; Simon et al., 2019). Fish activity was monitored after induction and upon visible excitation or when first eggs were found at the bottom of the tank, fish were fished. Females were processed first to obtain eggs: they were quickly blotted on a moist paper towel and laid on their side in a petri dish. They were gently but firmly maintained there while their flank was gently stroked. If eggs were not released immediately, the female was put back in the tank. Once eggs were collected, a male was quickly processed similarly to females, on the lid of the petri dish to collect sperm. The sperm was then washed on the eggs with 10–20 mL of tank water (conductivity ∼500 µS) and left for a few moments (30 s to 2 min, approximatively), after which embryo medium was added in the petri dish. Fertilised eggs were quickly laid on a zebrafish injection dish containing agarose grooves. They were injected with a Picospritzer III (Parker Hannifin) pressure injector.

### CRISPR injections and knock-in lines

sgRNA were designed to target the low-conservation regions between elements 1 and 2 and between elements 3 and 4. Two sgRNA were initially designed per region and sgRNA2 was found to efficiently cut the targeted region (Fig. S8**)**. The mix contained Cas9 protein generously provided by TACGENE and sgRNA2 with the following targeting sequence: CCCAATTCACCAGTATACGT (synthesised with AMBION T7 MEGAshortscript™ T7 transcription kit). Concentrations were kept with a 1:1.5 Cas9 to sgRNA molar ratio and varied between 0.71 µM (25 ng/µl) and 5.67 µM (200 ng/µl) of sgRNA 2, mostly 2.84 and 1.42 µM were used. The donor construct contained a HSP70 promoter used as a minimal promoter, a GFP cDNA and SV40 poly-adenylation signal, flanked by I-SceI meganuclease cutting sites. I-SceI was used to generate sticky ends and was either detached by 7 min at 96°C or injected with the construct. Concentrations of the repair construct varied between 3.33 and 10.92 nM but were mostly used at 10.71 nM.

Excellent *Zic1* pattern recapitulation in F0 was observed at low frequency (1–2% of injected embryos), and more partial patterns were more frequent. All potential founders were raised until males were sexually mature (6 months old) and could be screened by individual IVF. We obtained three SF founders (out of 15 F0 males screened) and 5 CF founders (out of nine screened), with good to excellent transmission rates: 4%, 7% and 30% for SF founders and 4%, 45%, 48%, 50% and 54% for CF founders, respectively. Fish were screened based on their GFP pattern, matching *Zic1* (Fig. 1E). In both morphs some variations in relative fluorescence intensities were observed, with some lines exhibiting homogeneous expression levels and others showing strong GFP fluorescence in the telencephalon and dimer fluorescence in the eye. We focused on the most homogeneous lines for imaging purposes.

### mRNA injection

Transgenic embryos used for live imaging were injected in the cell or yolk at 1 cell stage with a H2B-mCherry fusion mRNA at a concentration of 50 ng/µl.

### Imaging

Transgenic embryos were obtained by IVF with wild-type eggs and transgenic sperm and were immediately injected with H2B-mCherry mRNA for nuclear labelling. Injected embryos were screened for GFP and mCherry fluorescence under a Leica M165C stereomicroscope around 10–11 hpf, when GFP reporter fluorescence first becomes detectable.

Selected embryos were immediately mounted in a phytagel tube (Sigma-Aldrich, CAS Number: 71010-52-1) moulded with Phaseview Teflon mould (1.5 mm of diameter) and maintained in position with 0.4% low melting point agarose (Invitrogen UltraPure™ Low Melting Point Agarose). The tube containing the embryo was placed horizontally into the chamber containing 0.04% Tricaine in embryo medium (Sigma-Aldrich, CAS Number: 886-86-2). The tube was rotated under the microscope so that the embryo would face the objective.

Live imaging was performed approximately from 10.5–11 hpf to 24 hpf every 2.5–3 min, using a Phaseview Alpha^3^ light sheet apparatus, coupled with an Olympus BX43 microscope and using either a 20X/NA 0.5 Leica HCX APO objective or a 20X/NA 0.5 Olympus objective. Images were acquired using QtSPIM software (Phaseview), which controlled a Hamamatsu ORCA-Flash4.0 Digital sCMOS camera.

Room temperature was maintained at 24°C by air conditioning and the chamber temperature was further controlled by a BIOEMERGENCES-made thermostat. Medium level was maintained by a home-made perfusion system and an overflow to renew the medium. The orthogonal illumination of the SPIM induced minimal photo-damage, and embryos developing for more than 20 h under the microscope were alive with a normal head shape at 48–60 hpf, even though the tail was usually twisted due to the mechanical constraint in the low-melting agarose.

### Movie analyses

#### Morphogenesis

Macroscopic analyses result from quantifications made on *n*=4 eyes for each morph. Images were obtained and visualised with Arivis Vision4D software using re-oriented 3D stacks to allow similar optical section plane of analysis in different samples, cutting through the middle of the lens and the optic stalk at all time-steps. On one time-step per hour, measurements were performed on the re-oriented images: optic vesicle/optic cup length (at the widest), OV size increase (calculated by subtracting the length at the onset of furrow formation to the length at time t), optic stalk width, distance between the anterior optic cup and the lens, distance between the posterior optic cup and the lens, distance between the optic cup edges, position of the lens relative to anterior OV (=distance between centre of the lens and anterior OV/(distance between centre of the lens and anterior OV+distance between centre of the lens and posterior OV) (see schemes in Fig. 2 and Fig. S4).

#### Image stack treatments for cell tracking

Hyper-stacks used for tracking analyses were in 8-bit format. Pixel dimensions were 0.3 µm in x y, 1 µm in z, 39 t frames (2 min 30 sec each) and 420 and 360 z steps, respectively, for surface fish and cavefish embryo. To improve image quality and allow more convenient tracking in MAMUT, several image treatments were necessary. Pixel intensity of all images within each stack were homogenised using contrast enhancement (0.3%), and 3D drift correction to improve image alignment was performed. Image stack were registered in the H5 format.

#### Cell tracking

To study cell behaviours, we tracked cell nuclei during evagination, between 11.5 hpf and 13 hpf (1 h 40 min, 40 movie frames) using the FIJI plugin MAMUT (Schindelin et al., 2012; Wolff et al., 2018), which allowed identification of nuclei at each t frame in the 3D. Because of the threefold increased voxel size compared to x and y, nuclei appeared distorted in the z plane. We preferentially – but not exclusively – tracked nuclei of high fluorescence intensity, which greatly facilitated non-ambiguous nuclei tracking. All nuclei tracks used for trajectory analyses were meticulously analysed and checked twice.

For trajectory analyses, the (x,y,z) cell coordinates were extracted using MAMUT and distances in 3D or 2D (x,y) between time points were calculated using the Pythagoras formula. We used x,y,z coordinates to calculate cumulative distance and absolute distance in space covered in 3D as well as instantaneous migration speeds (distance covered/150 s). For the trajectory aspect, we used x,y coordinates to calculate instantaneous deviation angle at each time point using the Al-Kashi formula, valid in any triangle ABC, which relates the length of the sides using the cosine of one of the angles of the triangle. We calculated the value of the angle AB^AC in a triangle ABC, in which AB, BC and AC sides represent the distances covered by a nucleus between (t-t+1), (t+1-t+2) and (t-t+2), respectively. AB^AC=DEGRES(ACOS(((BC^2^)-(AB^2^)-(AC^2^))/-(ACxBC/2))).

To study proliferative activity, we tracked metaphases and anaphases manually and exhaustively in the whole brain/head of one SF and one CF embryo. To count mitotic events in OVs and presumptive lens without errors, each mitosis tracked and labelled in MAMUT was re-checked and allocated manually to structures or regions of interest (ROI) (see Fig. S6). Results were expressed either as absolute cell counts or normalised and expressed as densities to account for the difference of OV size between SF and CF (see Fig. S6). Two types of normalizations were applied, which lead to the same conclusion. First, the mitoses counts were normalised to the OV volumes, calculated on the movies using the plugin MZstack at 11.5, 12.5 and 13.5 hpf and averaged (Fig. 3E). Second, the mitoses counts were performed on maximum projections inside ROIs of identical size, in the OVs or in the medial neural tube as a control (see Fig. S6). In the case of the OV ROI, and because the optic vesicles are smaller in x,y but also in z (depth) in CF, a normalisation factor was applied. In SF, OV cell divisions were tracked along a z extent of 145, while in CF cell divisions were tracked on a z extent of 100. The normalisation factor was therefore x1.45 (Fig. 3E). For this proliferation analysis, statistical comparison could not be provided as we studied one SF and one CF sample.

### Statistics

Statistical significance and *P*-values were calculated using non-parametric Mann–Whitney U-tests in R. No statistical method was used to predetermine sample size. The experiments were not randomised, and the investigators were not blind to the experiment during image analyses.

## Supplementary Material

Supplementary information
