# Supplementary material for: Eye morphogenesis in the blind Mexican cavefish
Source: Biol Open. 2021 Oct 28;10(10):bio059031. doi: 10.1242/bio.059031 (PMC8565469; doi:10.1242/bio.059031)
Supplement: Supplementary information [file biolopen-10-059031-s1.pdf]

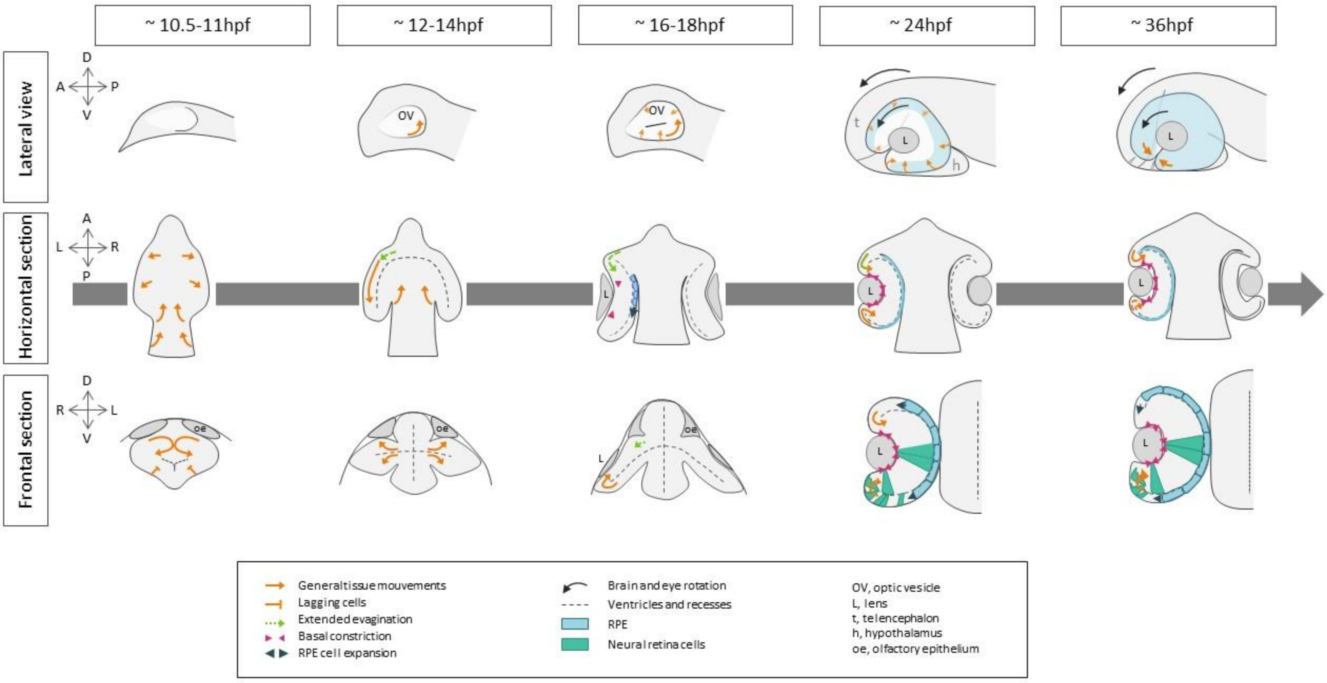

Fig. S1. Eye morphogenesis in fish.

Schemes depicting the principal steps of eye morphogenesis in fish models, summarized from the available literature cited in Introduction.

Stages and orientations are indicated.

Orange arrows show general cell and tissue movements.

Black arrows show the anterior-wise rotation of the eye and brain.

Green arrows show the contribution of extended evagination.

Pink arrowhead show cellular basal constriction.

The blue color depicts the RPE cells, while the green color depicts retina neuroepithelium cells changing shape.

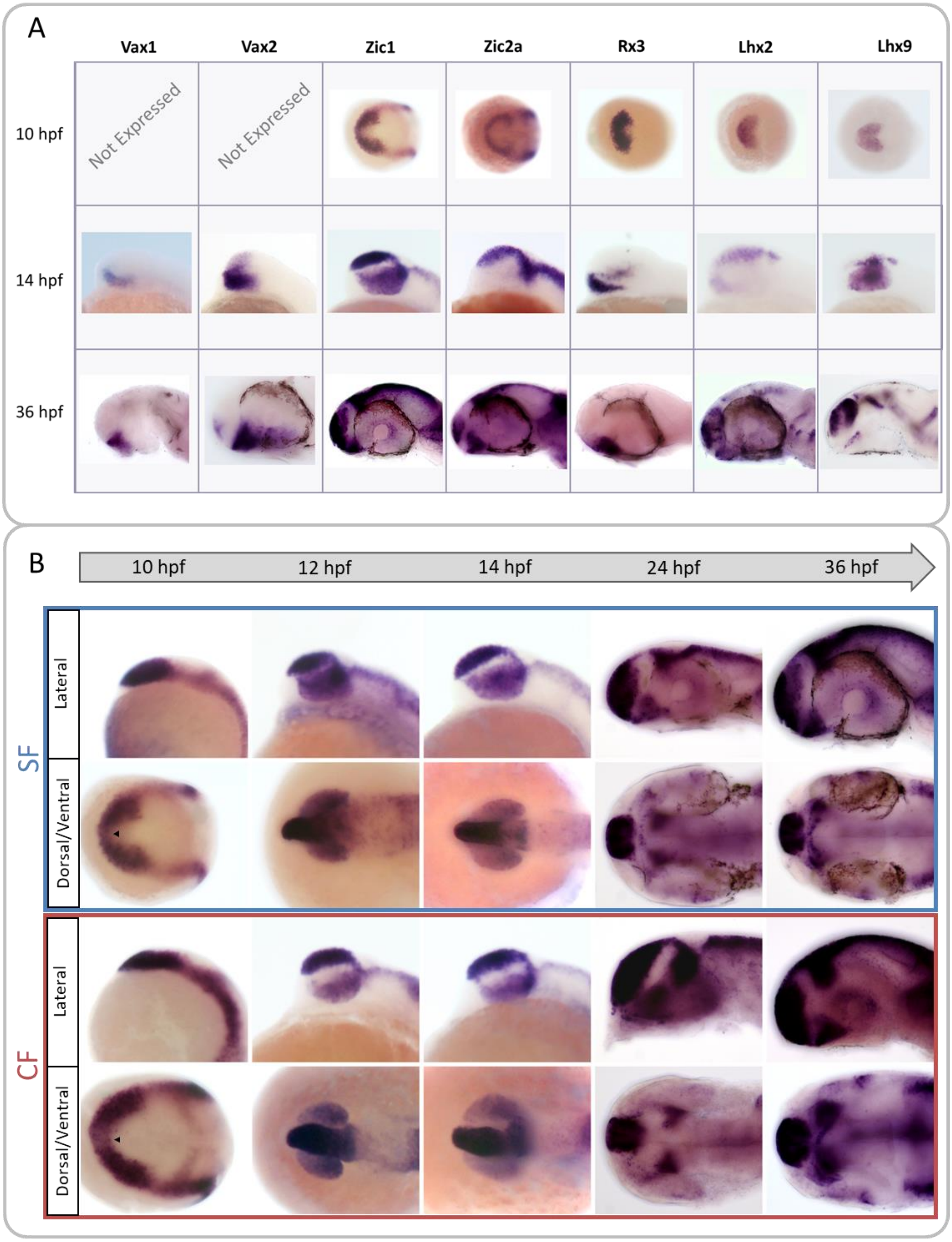

Biology Open • Supplementary information

Fig. S2. Choosing a candidate gene for transgenesis.

Chosen candidates were V*ax1, Vax2, Zic1, Zic2a, Rx3, Lhx2* and *Lhx9*. (A) Mini-screen of candidate genes by *in situ* hybridization at different stages (10, 12, 14, 24 and 36hpf) of interest on surface fish and cavefish (not shown) embryos. Anterior is to the left. Dorsal views at 10hpf; lateral views at 14hpf and 36hpf. The eyes were dissected out for *Vax1* and *Lhx9* (as no eye expression was detected for either of them) to allow better visibility of the inner tissue. Among the 7 genes, 5 were expressed in the anterior neural plate at 10hpf while 2 were not: *Vax1* and *Vax2*, whose expressions were detectable from 12hpf only. Five of them were expressed at least partially in the OV per se (excluding ORR and optic stalk): *Vax2*, *Zic1*, *Rx3*, *Lhx9* and *Zic2a* (faintly). At 36hpf, only 4 of them were still expressed in the optic cup: *Zic2a* and *Zic1* (around the lens), *Lhx2* (faintly) and *Vax2* (in the ventral retina). Subtle differences between CF and SF expression patterns were observed (not shown), and only one candidate gene was consistently expressed in the eye from neural plate to 36hpf: *Zic1.*

(B) Dtailed analysis of *Zic1* expression pattern at 5 different stages in surface (SF) and cavefish (CF). Anterior is to the left, at 10, 12 and 14hpf, bottom pictures are taken in dorsal view; at 24 and 36hpf, bottom picture are taken in ventral views. Arrowheads indicate an indentation in the eyefield.

Biology Open • Supplementary information

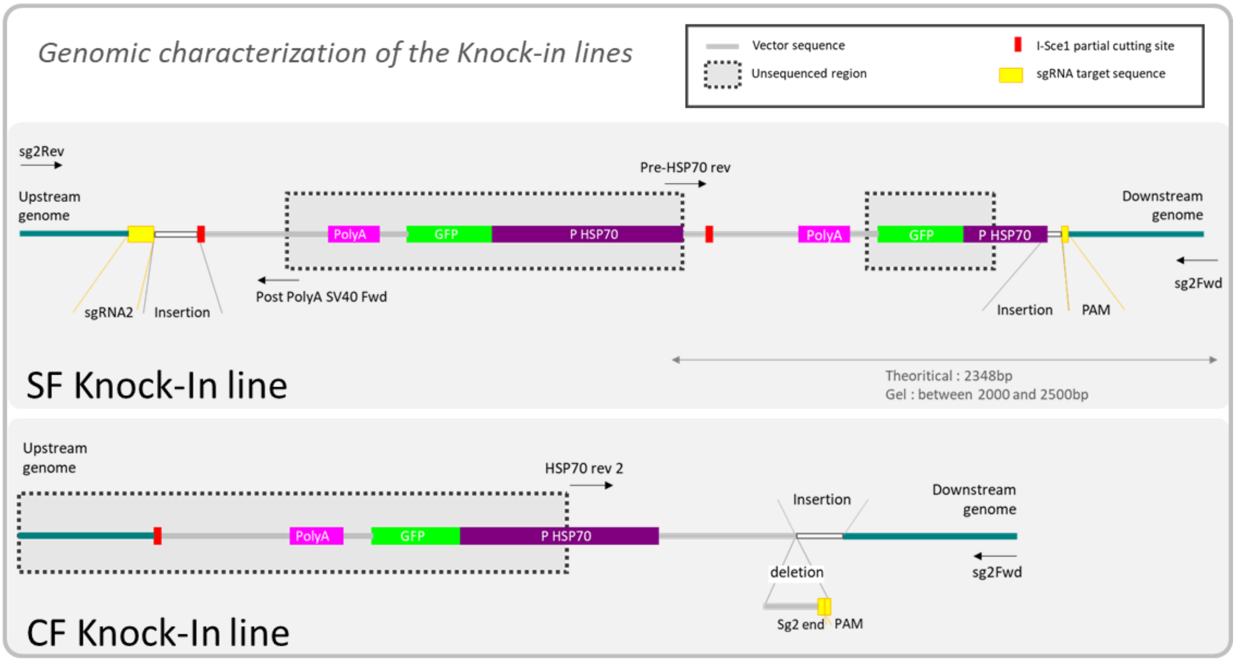

Fig. S3. Genomic characterization of the Knock-in lines.

Knock-In insertions, based on partial sequencing. Dotted boxes indicate un-sequenced regions, leaving uncertainties. For example, in the surface fish line, there is at least a partial insertion of the repair construct, containing a truncated Hsp70 promoter and at least another insert in the same direction (but potentially several). Of note, the surrounding genomic region is very rich in T and A (GC content around 35%) with many repeats, making PCRs sometimes challenging.

The data show that for both lines the transgenes are inserted at the correct targeted site.

Biology Open • Supplementary information

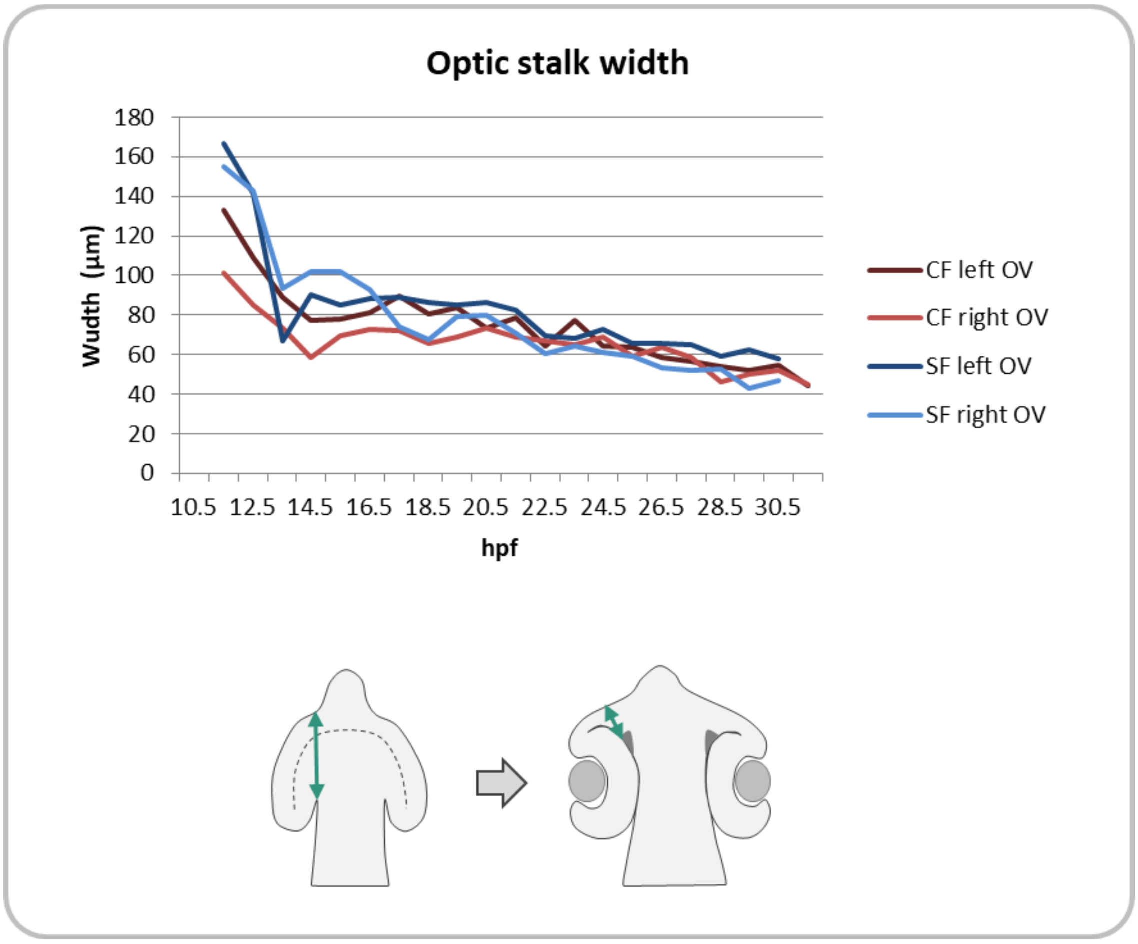

Fig. S4. Optic stalk width.

The size of the optic stalk (in a wide meaning: the connection between the OV and the neural tube) is smaller in cavefish during early development due to the smaller size of the OV but rapidly becomes indistinguishable from the optic stalk of the surface fish.

Biology Open • Supplementary information

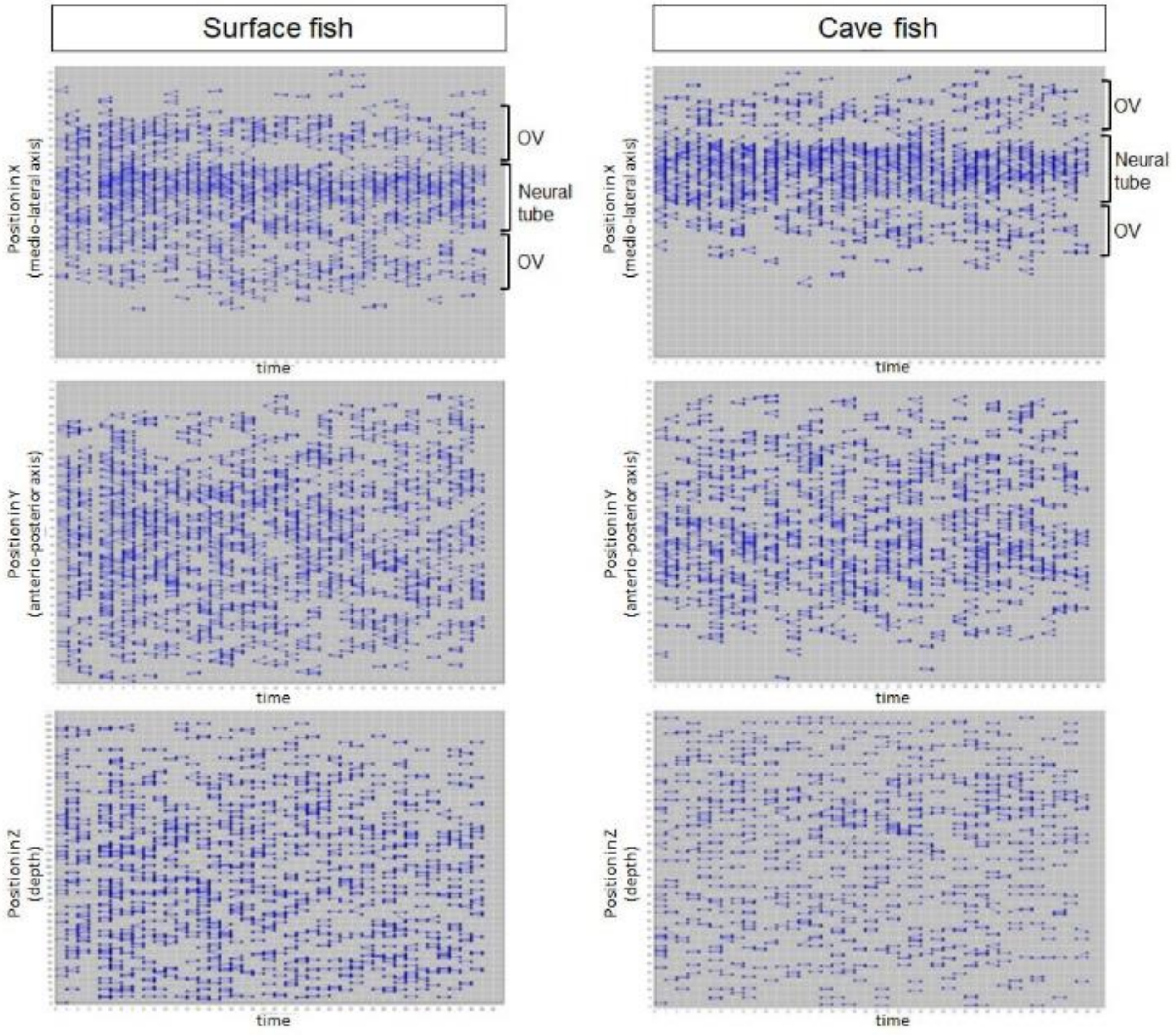

Fig. S5. Distribution of mitoses in the head and as a function of time, between 11.5hpf and 13hpf, in SF and CF embryos.

Plots showing the distribution of mitoses (schematically represented by a mother cell linked to daughter cells) in SF (left) and in CF (right). The 3 plots show the distribution of mitoses in X (medio-lateral axis), Y (antero-posterior axis) and Z (depth), as a function of time. Note the homogeneous repartition of divisions in the tissue, including in Z, suggesting that mitoses could be properly tracked, even in the depth of the tissue.

Biology Open • Supplementary information

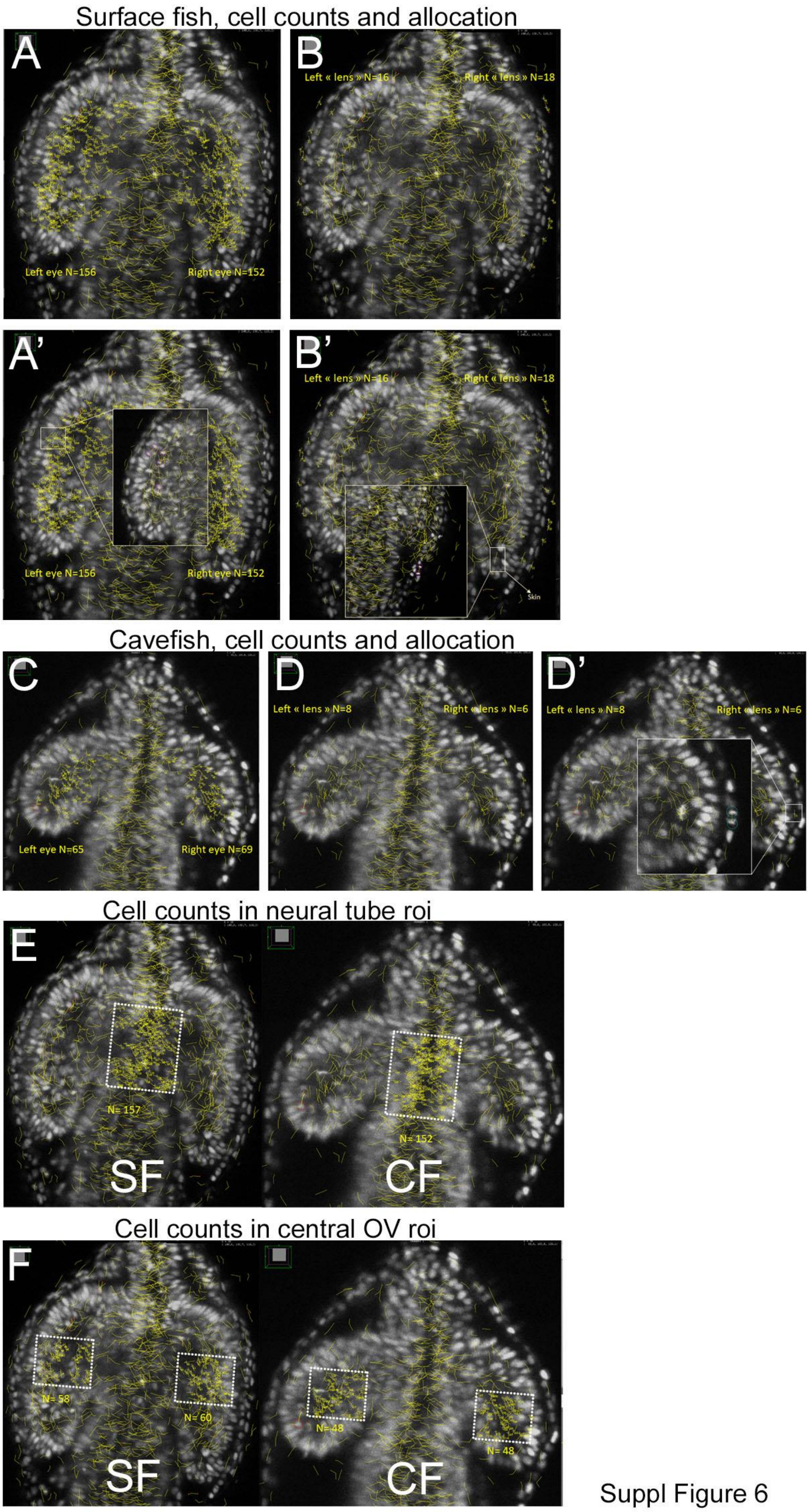

Suppl Figure 6

Biology Open • Supplementary information

Fig. S6. Counting mitoses, and normalization.

(A-D’) Illustration of the method used to count mitoses in SF (A-B’) and CF (C-D’). To count mitoses in OVs and presumptive lens without errors, each mitosis tracked and labelled in MAMUT/Fiji was re-checked and segmented manually for proper allocation. Insets in A’B’D’ show examples of cells that appear like they belong to the OV region on the maximum projection, but that were attributed either to the OV, the skin or the lens after manual re-segmentation.

(E) Illustration of mitosis counts in a medial neural tube roi of the same size in SF and CF, for estimation of mitotic density in the tissue.

(F) Illustration of cell counts in OV roi of the same size in SF and CF for estimation of mitotic density in the tissue. Here, because the CF optic vesicles are smaller in XY but also in Z (depth), a normalisation factor was applied. In SF, OV cell divisions were tracked along a Z extent of 145, while in CF cell divisions were tracked on a Z extent of 100. The normalisation factor was therefore x1.45 (**Fig. 3E**).

Biology Open • Supplementary information

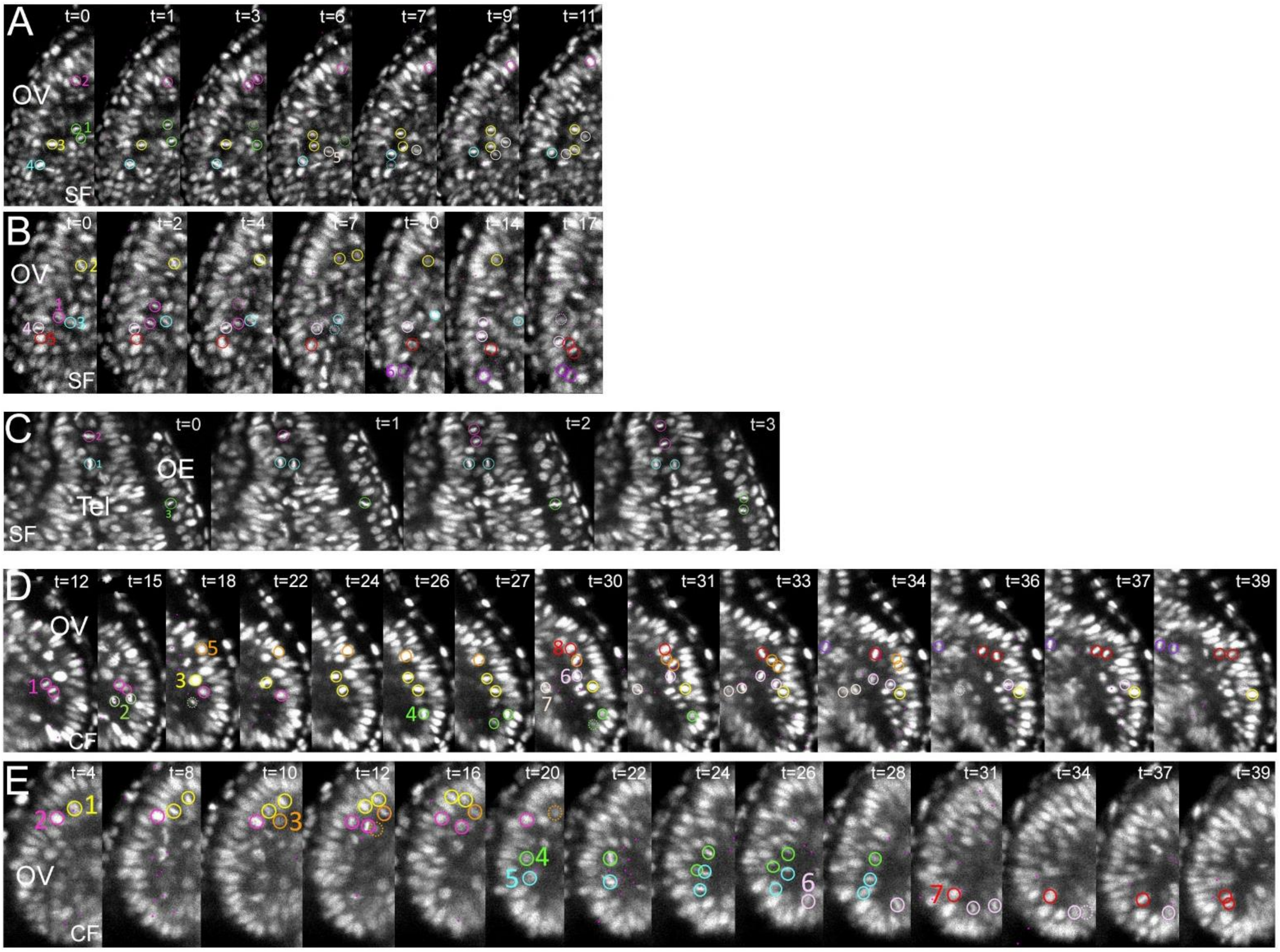

Fig. S7. Additional examples of mitotic behaviors at high power magnification, with comments.

Cell nuclei are labelled with colored circles; numbers indicate the order in which tracked cells will divide; daughter cells that migrate and that are lost in Z in the plane shown are indicated by dotted circles.

(A,B) show the same cell divisions sequence as in Figure 3, in the SF OVs. In A: The pink cell (#2) divides along the ventricle in the anterior OV and its daughter cell migrates and rapidly integrates in the neuroepithelium. The green and the beige cells (#1 and #5) divide in the proximal side of the ventricle and their daughter cells move towards the inner leaflet of the OV. So does the yellow cell (#3), although its initial position is more distal. Note the rotation/orientation behavior of the metaphasic plate of the blue cell, before dividing (#4).

(C) shows cell divisions in the telencephalon (Tel) and the olfactory epithelium (OE) of SF. The pink and the blue cells (#1 and 2) divide in the telencephalon along the ventricular border,

Biology Open • Supplementary information

with orthogonally-oriented metaphasic plates. The green cell (#3) divides in the olfactory epithelium.

(D,E) show cell divisions sequences in the CF OVs. D is the same sequence as in Figure 3. In D: the pink (#1), yellow(#3), rose (#6) and red (#8) cells are representatives of all those cells that divide at the ventricle and then rapidly migrate to incorporate in the neuroepithelium. The orange cell (#5) follows a typical complete sequence: delamination from neuroepithelium, division at the ventricle, and reintegration of daughter cells back in the neuroepithelium. The kaki (#2) and the beige (#7) cells divide and populate the inner leaflet of the OV. The purple cell divides at the level of the optic recess region (ORR).

Biology Open • Supplementary information

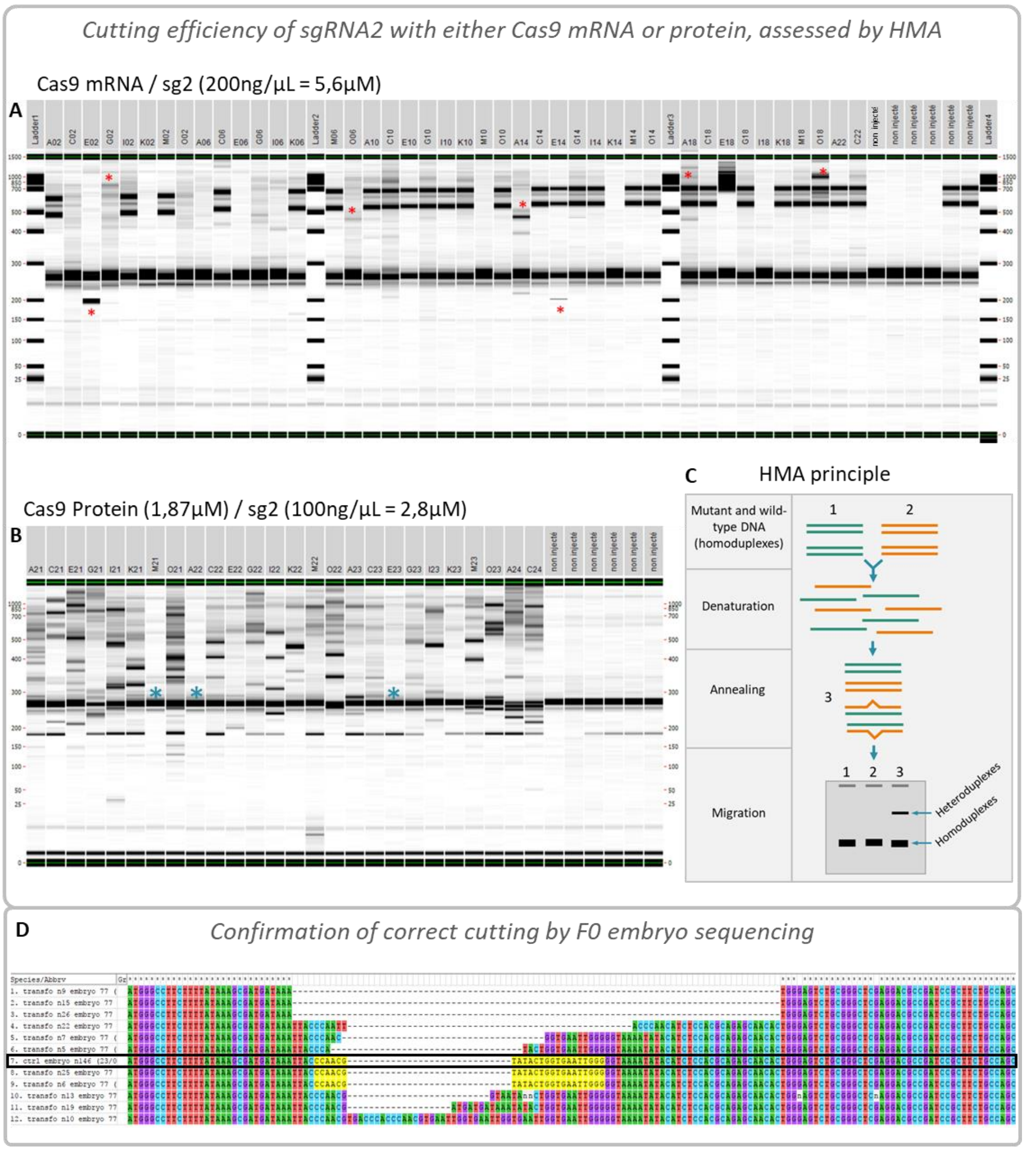

Fig. S8. Cutting efficiency of sgRNA 2

(A) Assessment of sgRNA 2 cutting efficiency when injected with Cas9 mRNA by heteroduplex mobility assay (HMA, explained in (C)). Each column is an individual F0 embryo. Embryos with strong additional bands are labelled with a red asterisk; additional light bands can be seen in several individuals, indicating cuts and imprecise repairs. Note that the 2 heavy bands seen on many embryos are also present in some of the un-injected controls (the 6 columns on the right) indicating a polymorphism in this region in the wild-type fish (not on the sgRNA target sequence).

(B) Assessment of sgRNA 2 cutting efficiency when injected with Cas9 protein, note the strong presence of additional band compared to the 6 control embryos on the right. Embryos without

Biology Open • Supplementary information

any visible cuts are labelled with a blue asterisk. Additional bands are seen much more frequently and are much more important than with the Cas9 mRNA injection, probably indicating more frequent but also more precocious cut and repair events in the embryo, so that many cells share the same sequence.

(C) Heteroduplex mobility assay: in an electrophoresis, heteroduplexes are slowed down compared to homoduplexes so that they form additional bands that can be seen even if the polymorphism is only a single substitution. In short, the DNA fragments are denatured and renatured to form heteroduplexes. An electrophoresis is then performed (here with a LabChip, PerkinElmer) to detect the presence of polymorphism.

(D) Different cutting and repair events in a single injected embryo. A PCR was performed on one injected embryo (100ng/µL sgRNA2, Cas9mRNA) around the sgRNA2 target site and the product was cloned. Plasmidic preparations from individual colonies were then sequenced. Various sequences were obtained, evidencing different cut and repair events in one single embryo. sgRNA2 target sequence is highlighted in yellow whenever intact. This F0 fish harboured both insertions and deletions around the cutting site of sgRNA2. A non-injected control fish sequence is included, outlined in black.

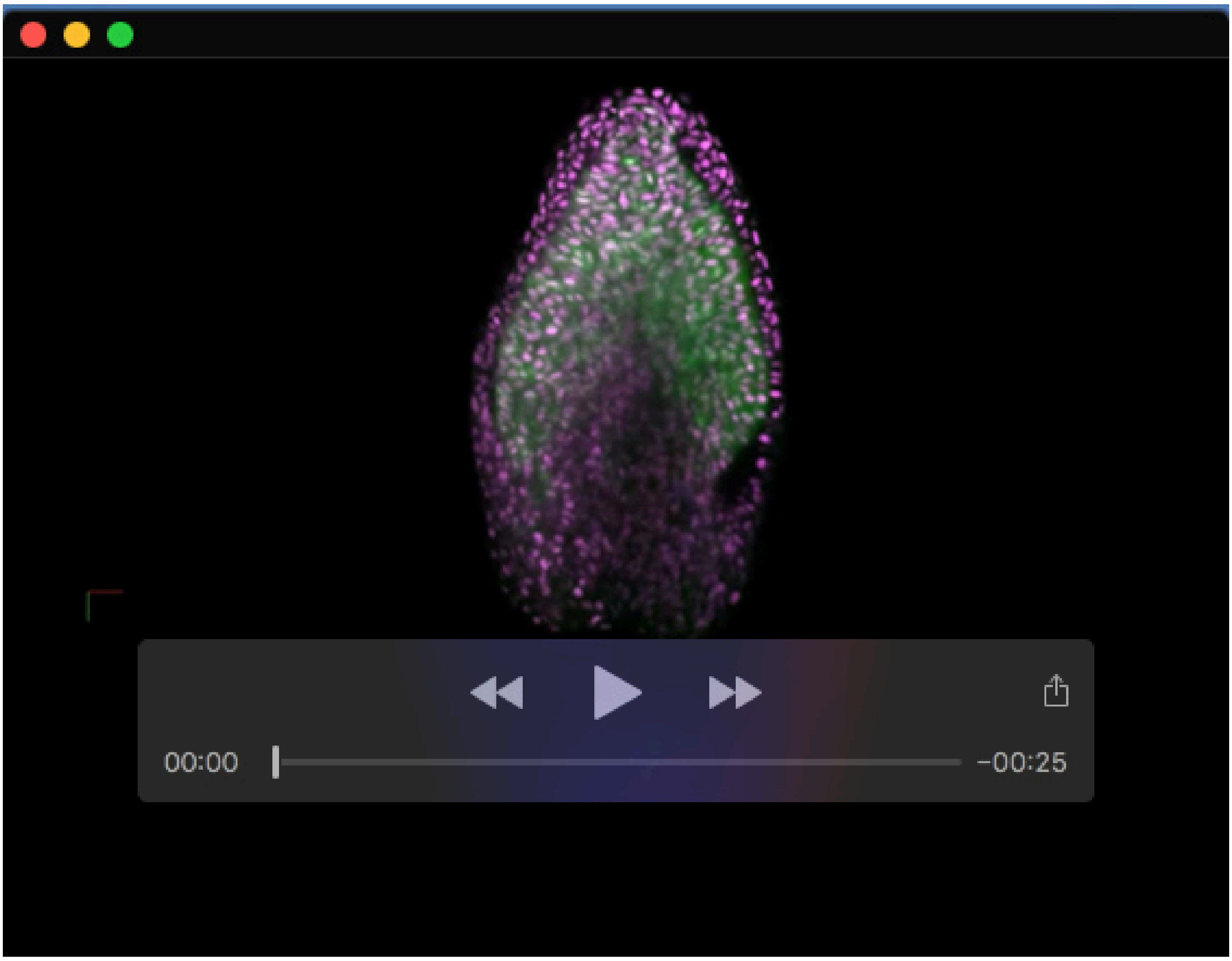

Movie 1.

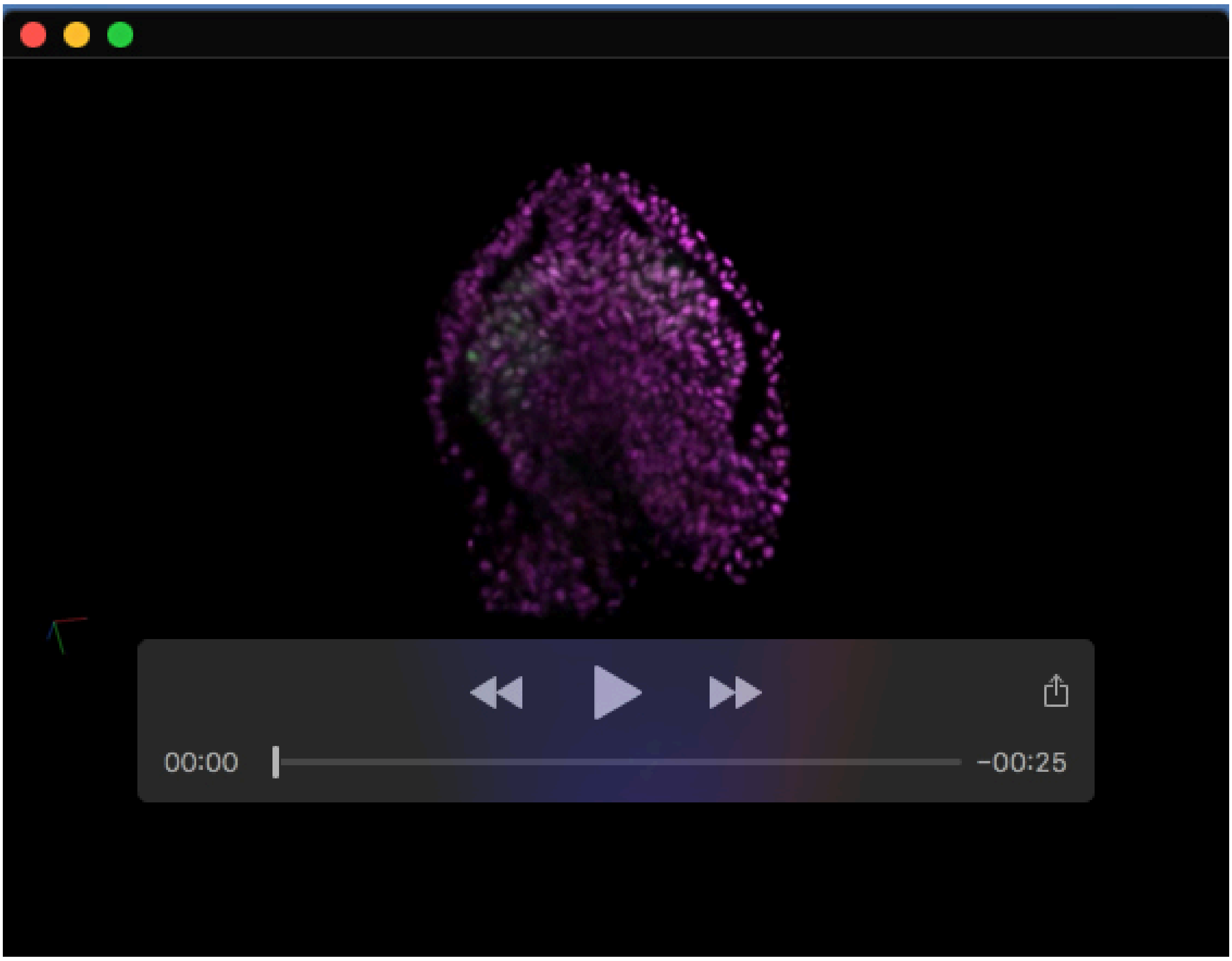

Movie 2.

Biology Open • Supplementary information

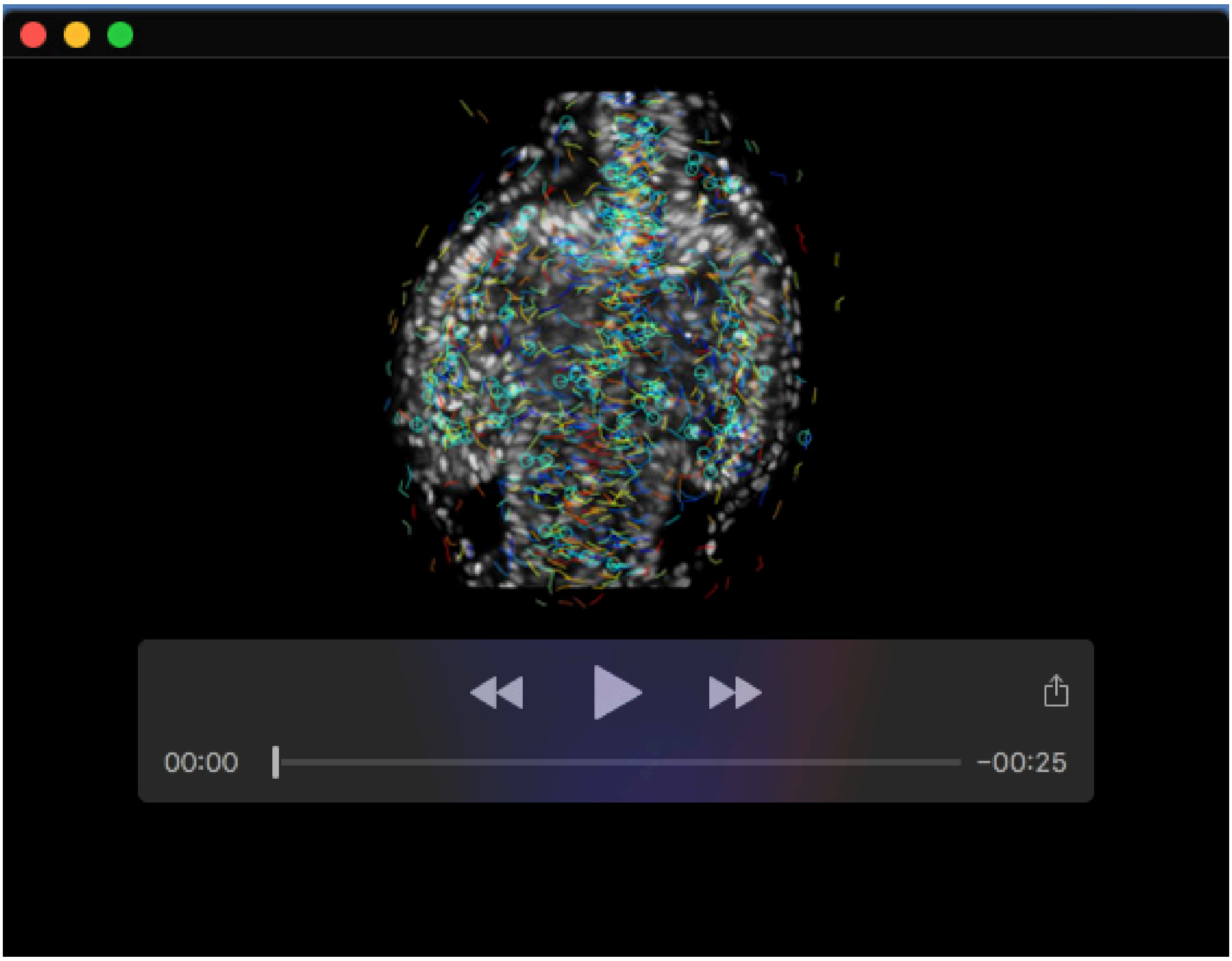

Movie 3.

Biology Open • Supplementary information

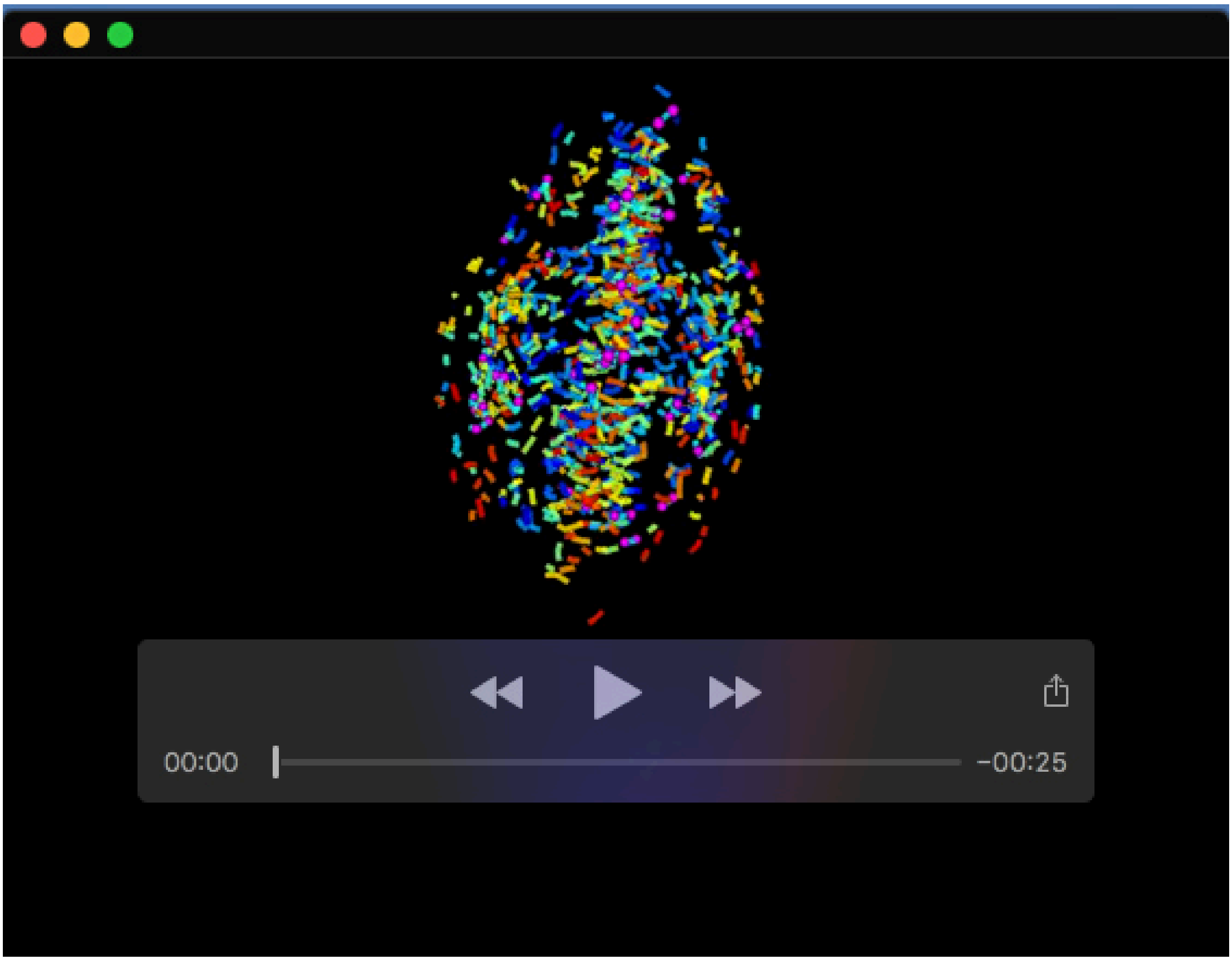

Movie 4.

Biology Open • Supplementary information

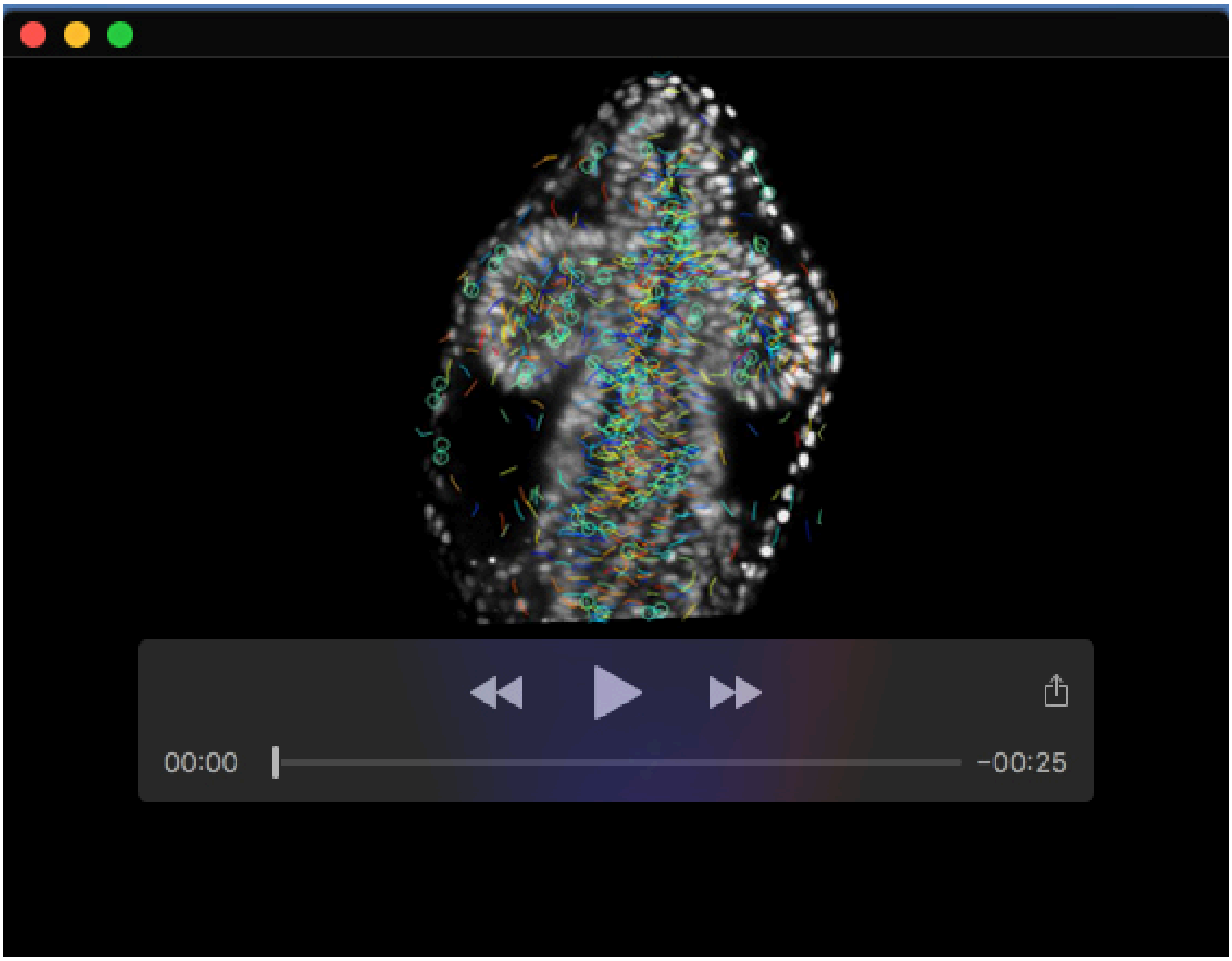

Movie 5.

Biology Open • Supplementary information

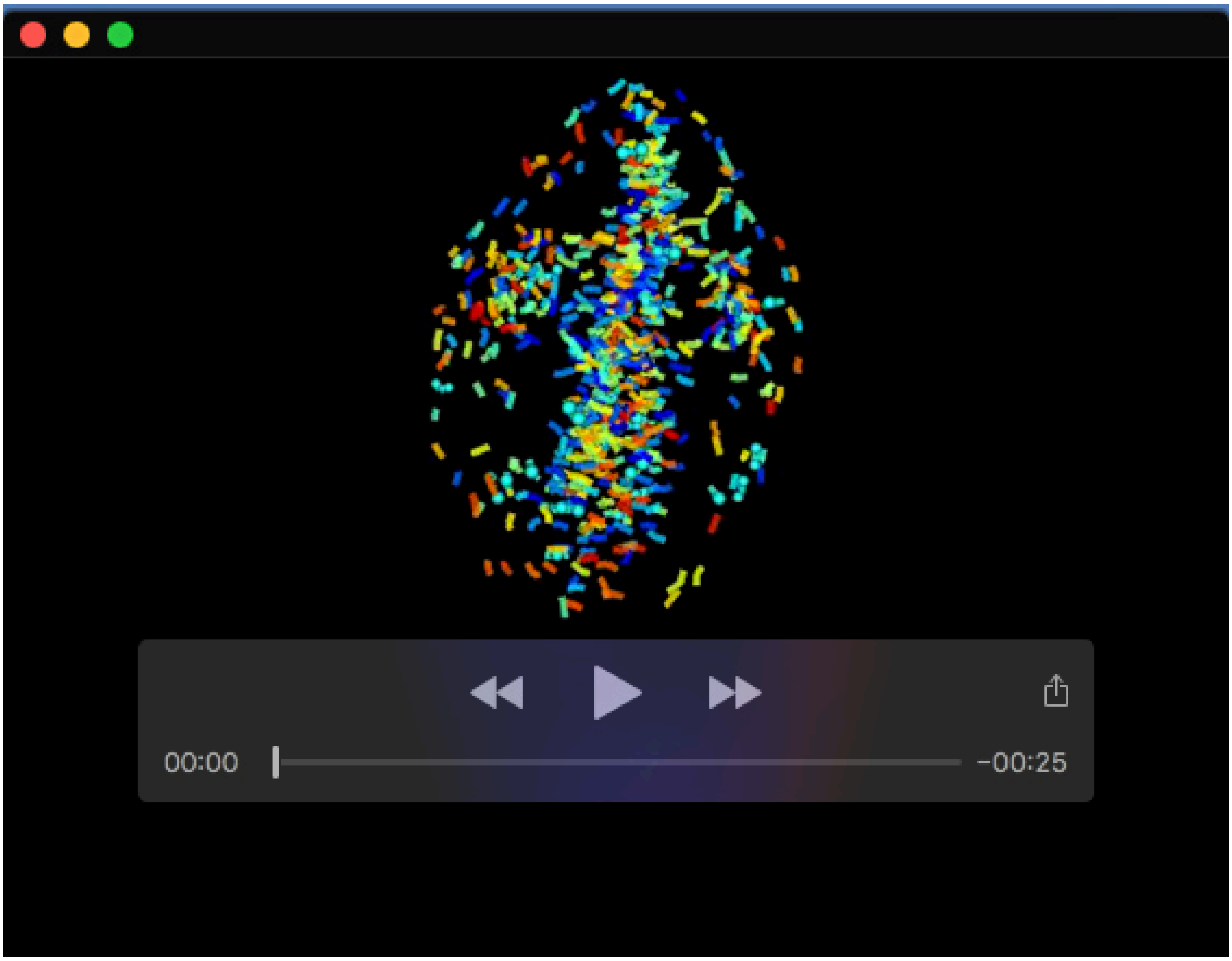

Movie 6.

Biology Open • Supplementary information

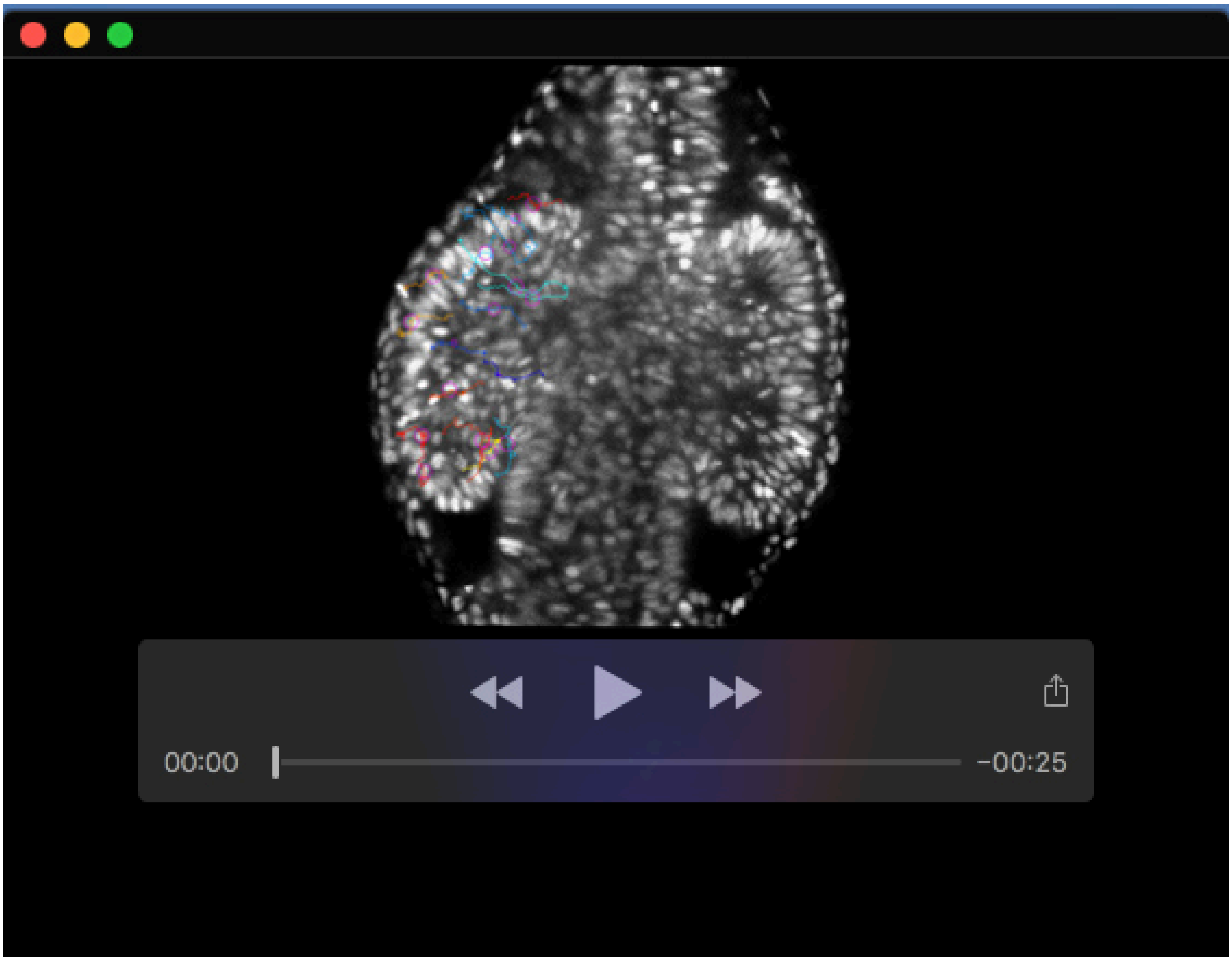

Movie 7.

Biology Open • Supplementary information

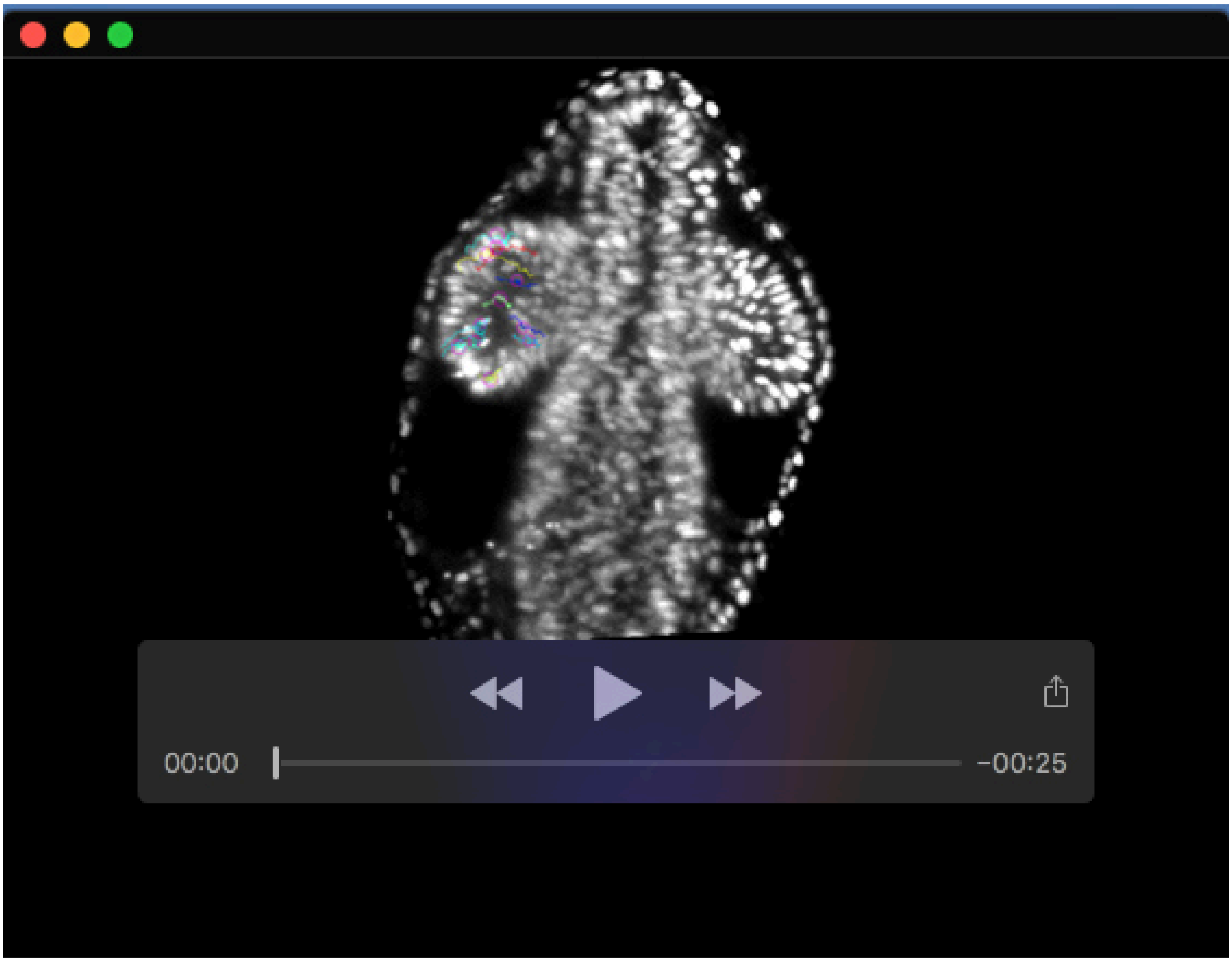

Movie 8.

Biology Open • Supplementary information
